# The MEF2A transcription factor interactome in cardiomyocytes

**DOI:** 10.1038/s41419-023-05665-8

**Published:** 2023-04-05

**Authors:** Amira Moustafa, Sara Hashemi, Gurnoor Brar, Jörg Grigull, Siemon H. S. Ng, Declan Williams, Gerold Schmitt-Ulms, John C. McDermott

**Affiliations:** 1 Department of Biology, York University, Toronto, ON M3J 1P3 Canada; 2 Muscle Health Research Centre (MHRC), York University, Toronto, ON M3J 1P3 Canada; 3 Centre for Research in Biomolecular Interactions (CRBI), York University, Toronto, ON M3J 1P3 Canada; 4 Analytical Sciences, Sanofi, Toronto, ON M2R 3T4 Canada; 5 Department of Mathematics and Statistics, York University, Toronto, ON M3J1P3 Canada; 6 Tanz Centre for Research in Neurodegenerative Diseases, and Department of Laboratory Medicine and Pathobiology, University of Toronto, Toronto, ON M5T 0S8 Canada; 7 Present Address: Seneca College, School of Health Sciences, King City, ON L7B 1B3 Canada; 8 Present Address: Analytical Development, Notch Therapeutics, Toronto, ON M5G 1M1 Canada

**Keywords:** Transcriptional regulatory elements, Protein-protein interaction networks

## Abstract

Transcriptional regulators encoded by the Myocyte Enhancer Factor 2 (MEF2) gene family play a fundamental role in cardiac development, homeostasis and pathology. Previous studies indicate that MEF2A protein-protein interactions serve as a network hub in several cardiomyocyte cellular processes. Based on the idea that interactions with regulatory protein partners underly the diverse roles of MEF2A in cardiomyocyte gene expression, we undertook a systematic unbiased screen of the MEF2A protein interactome in primary cardiomyocytes using an affinity purification-based quantitative mass spectrometry approach. Bioinformatic processing of the MEF2A interactome revealed protein networks involved in the regulation of programmed cell death, inflammatory responses, actin dynamics and stress signaling in primary cardiomyocytes. Further biochemical and functional confirmation of specific protein-protein interactions documented a dynamic interaction between MEF2A and STAT3 proteins. Integration of transcriptome level data from MEF2A and STAT3-depleted cardiomyocytes reveals that the balance between MEF2A and STAT3 activity exerts a level of executive control over the inflammatory response and cardiomyocyte cell survival and experimentally ameliorates Phenylephrine induced cardiomyocyte hypertrophy. Lastly, we identified several MEF2A/STAT3 co-regulated genes, including the MMP9 gene. Herein, we document the cardiomyocyte MEF2A interactome, which furthers our understanding of protein networks involved in the hierarchical control of normal and pathophysiological cardiomyocyte gene expression in the mammalian heart.

## Introduction

Cardiac muscle development and post-natal adaptations are accompanied by transcriptional activation of a battery of structural genes encoding components of the contractile machinery, enzymes, receptors, and ion channels. Moreover, transcriptional circuits in the post-natal vertebrate heart are known to play a role in maladaptive cardiac hypertrophy and remodeling in heart failure progression [1, 2]. Cardiac hypertrophy often precedes heart failure [3] and is initially a physiologic growth response of the heart resulting from increased mechanical loading due to high blood pressure or myocardial damage caused by ischemic heart disease. In addition to hypertrophy of cardiomyocytes (CMs), the progression to HF also includes an irreversible loss of CMs due to cell death pathways such as apoptosis, necrosis and necroptosis [4, 5]. Congenital disorders of structural and contractile proteins of the myocardium can also lead to hypertrophy, cell death and eventual heart failure [6]. Disease of the adult heart thus activates a pathological transcriptional response characterized by activation of cellular pathways leading to changes in gene expression underlying abnormal growth and death of CM’s. These pathological events, as well as normal cardiac ontogeny and physiology, are primarily accomplished by transcriptional regulators [7, 8]. The assembly and regulation of transcriptional regulatory protein complex networks in a CM are thus a primary determinant of its gene expression signature and phenotype. A number of transcription factors such as GATA4, NKX2-5 and MEF2 have been previously identified as playing important developmental and post-natal roles in determining the cardiac transcriptome [9–12]. Thus, understanding the transcriptional regulators that control myocardial gene expression is a predominant theme in understanding the molecular control of ontogeny, physiology and pathology of the mammalian heart.

Extensive work concerning the control of cardiac specific gene expression [1, 13–15] and also loss of function analysis in gene targeted mice [12, 16, 17] has positioned Myocyte Enhancer Factor 2 transcriptional regulatory proteins (MEF2) as important regulators of cardiac gene expression during development and in post-natal regulation of the heart through adulthood [14, 18–20]. MEF2 has also been implicated as a fundamental regulator of the hypertrophic response of cardiomyocytes in response to pressure overload, indicating its potential role in pathology of the adult heart [2, 21].

The MEF2 family of transcription factors (encoded by four genes labeled MEF2A to D) have proved crucial in regulating cardiac [22], skeletal [13, 18], and smooth muscle differentiation [23], neuronal survival [24, 25], and T cell activation [26]. The MEF2s belong to the MADS (MCM1, Agamous, Deficiens and SRF) superfamily of DNA binding proteins. The amino terminus of MEF2 proteins is highly conserved among all family members and consists of a 57 amino acid MADS domain [27] and a 29 amino acid MEF2 domain that mediates MEF2 protein dimerization, cofactor interaction and binding to a cognate *cis* element with the consensus (T/C)TA(A/T)_4_TA(G/A) [28–31]. The MEF2 transactivation domain (TAD) is located in its carboxyl-terminus and the sequence in this region is more divergent between different MEF2 family members [13]. The TAD is subjected to extensive alternative splicing and posttranslational modification by phosphorylation, acetylation and sumoylation [32–36]. MEF2s have been shown to be highly responsive to several signal transduction cascades, and their post-translational regulation by covalent modification by PKC [34], p38 MAPK [34, 37, 38], ERK5 [39, 40] and PKA have been well documented by ourselves and others [41–43]. Previous work indicates that the diversity of roles carried out by MEF2 proteins, as alluded to above, can be partially explained by post-translational modifications and extensive protein-protein interactions that modulate its function depending on the prevalence of tissue specific partners and signaling pathway activation. In skeletal muscle, MEF2 association with the muscle regulatory factors (MRFs) is a well characterized paradigm for control of skeletal myogenesis [30] whereas an analogous MEF2 partner has not so far been identified in cardiac muscle. Unlike the interaction with MRFs, which is highly tissue specific and transcriptionally synergistic, MEF2 interaction with class IIa HDACs, such as HDAC4, is well documented in several biological systems and results in repression of MEF2-dependent transcription through direct HDAC interaction with the amino terminal MADS/MEF2 domain [44]. In terms of cardiac gene expression, MEF2A is of particular interest since MEF2A homozygous null mice are prone to perinatal sudden death within a week due to heart failure, characterized by ventricular dilation, myofibrillar disorganization and disordered mitochondria [16]. Due to the predominant but partial penetrance of the lethal phenotype in these mice, some surviving MEF2A null mice exhibited mitochondrial deficiency and were susceptible to sudden death [16]. This variable penetrance also suggests the possibility of an overlap in function with other MEF2 factors expressed in cardiomyocytes.

In view of the fundamental and essentially distinct role played by MEF2A in cardiac muscle development, homeostasis and pathology, we endeavoured to characterize its protein interaction network (interactome) in cardiomyocytes. To do this we undertook a systematic unbiased screen of the MEF2 interactome in cardiomyocytes using affinity purification coupled with quantitative proteomics, based on the idea that interactions with regulatory protein partners underly the diverse roles that MEF2 exerts in the control of cardiomyocyte gene expression. Coupled with transcriptome analysis, this strategy has allowed identification of key MEF2 protein interactions in primary cardiomyocytes, revealing potential roles in regulation of programmed cell death, inflammatory responses, actin dynamics and stress signaling. Further biochemical and functional confirmation of an identified interaction between MEF2A and the STAT3 protein was deemed of particular interest due to both factors being previously identified independently as key regulators of cardiomyocyte gene expression. Here, we present evidence that the balance and interaction between MEF2 and STAT3 proteins exerts a level of executive control over several important biological processes in mammalian cardiomyocytes.

## Results

### Identification of the MEF2A interactome in primary cardiomyocytes

**Fig. 1:**
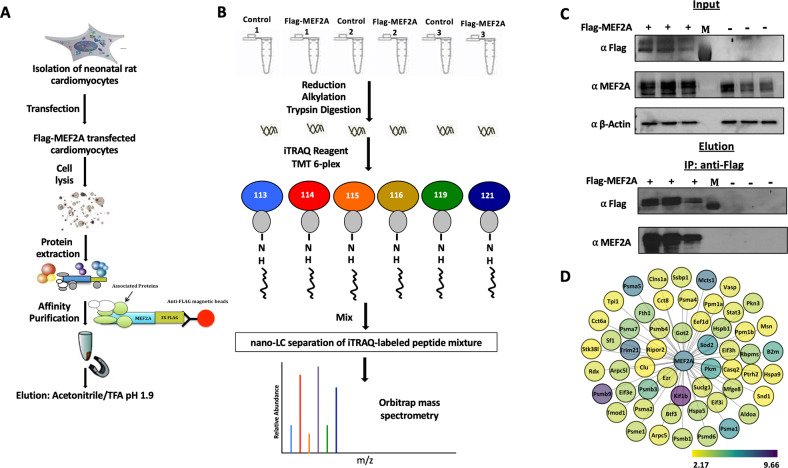
Affinity purification and qualitative proteomic strategy to identify the Flag-MEF2A protein complex in PCMs. **A** PCMs are first isolated and then transfected with a 3xFlag-MEF2A construct. The Flag-MEF2A complex was pulled down and purified using α-Flag magnetic beads. The beads were washed several times and the Flag-MEF2A complex was eluted with Acetonitrile/TFA pH 1.9. **B** Six independent samples of Flag-MEF2A lysates and non-transfected lysates were used to run six IPs. The elutes were trypsin digested and then labelled with iTRAQ reagents. Isobaric labelling reagents that produce reporter ions at m/z 117 and 118 were not used. The labeled peptides were separated using HPLC and then identified using MS/MS by relative quantification. **C** 3xFlag MEF2A and non-transfected lysates were subjected to Western blotting to indicate protein expression (Top panels). Six separate IP experiments were performed with α-Flag-magnetic beads then eluted and blotted with anti-Flag and MEF2A antibodies to confirm the AP step. IP with lysates from non-transfected PCMs were used as negative controls. M is an indication of the loading of the marker lane (Bottom panels). **D** 56 proteins in the Flag-MEF2A complex identified by mass spectrometric analysis. The node color corresponds to fold enrichment of the Flag-MEF2A interacting proteins in the three replicates, as defined in the legend. The node position and edge length are arbitrary. The bait (Flag-MEF2A) is represented inside a black square.

To identify MEF2A protein interacting partners, primary neonatal rat ventricular cardiomyocytes (PCMs) were prepared using ~50 neonatal hearts for each condition (50 hearts were processed for Flag-MEF2A and 50 hearts for the corresponding control conditions). Figure 1A illustrates the workflow for the experimental approach (see Methods for experimental details). The MEF2A protein complexes from the samples were captured using anti-Flag magnetic beads, followed by trypsin digestion, 6-plex iTRAQ peptide labelling and high pH reversed phase fractionation. We performed proteomic profiling of the Flag precipitated MEF2A complexes using quantitative proteomics as described in Methods and depicted in Fig. 1B.

Western blotting of the anti-Flag immunoprecipitates and corresponding lysates confirmed the transfection and immunoprecipitation. Firstly, Fig. 1C (top panel) documents the ectopic expression of Flag-MEF2A transfected PCMs compared to the negative control cells. Flag-MEF2A was detected (~72 KDa) in the transfected cells, while absent in the control samples. The next lower panel depicts western blots using a polyclonal antibody that detects MEF2A protein, indicating expression of the exogenous Flag-MEF2A in the transfected cells and also the endogenous MEF2A protein level in the PCMs. The third panel depicts Actin as the loading control in lysates. Figure 1C (lower) shows detection of Flag-MEF2A and total MEF2A in anti-Flag IPs using the transfected lysates. These data confirm successful enrichment of the Flag-MEF2A (detected by both anti-Flag and anti-MEF2A antibodies) and the absence of Flag-MEF2A in the corresponding negative controls for the mass spectrometry analysis.

Using a iTRAQ-based mass spectrometry analysis for the relative quantitation of proteins in our eluate fractions (see methods for details), we identified 56 proteins that co-enriched with Flag–MEF2A and as such represent the candidate interactome for this bait protein from PCMs (Table 1 supplementary document 1). As anticipated, the Flag-MEF2A bait protein was highly enriched in all MEF2A pull downs, reflected by 1642 high confidence peptide-to-spectrum matches (PSMs). Along with MEF2A, this approach identified PP1α, a previously documented MEF2A interacting protein [45]. Figure 1D illustrates the complete set of enriched proteins in the Flag-MEF2A IPs with their fold enrichment in the three Flag-MEF2A IPs.

### Gene ontology of the MEF2A interactome

To understand the biological context of our PCM interactome, a Gene Ontology analysis was employed to characterize the identified proteins. The enriched BP, MF, and CC GO terms are documented in supplementary document 1. Then, we used a functional annotation clustering tool to group relevant annotations and reduce the redundancy between them. This tool measures the degree of common genes between the annotations and classifies them into clusters with enrichment scores [46, 47]. The higher Enrichment scores are accompanied by a lower *p*-value of annotation terms (*p*-value < 0.1). The nine clusters with the highest score are identified in supplementary document 1. Functional annotation revealed that the MEF2A-interacting partners were enriched for actin-binding, regulation of gene expression, focal adhesion, glycolytic process, and translation initiation. We found that some genes, VASP, TMOD1, HSPA5, RDX, MSN, EZR, and STAT3, were involved in numerous functions such as actin-binding, focal adhesion, and positive regulation of gene expression. The results also revealed that TPI1, PKM, and ALDOA were enriched for the glycolysis process. Three genes, EIF3I, EIF3H, and EIF3E, were related to translation control.

### MEF2A protein network analysis

**Fig. 2:**
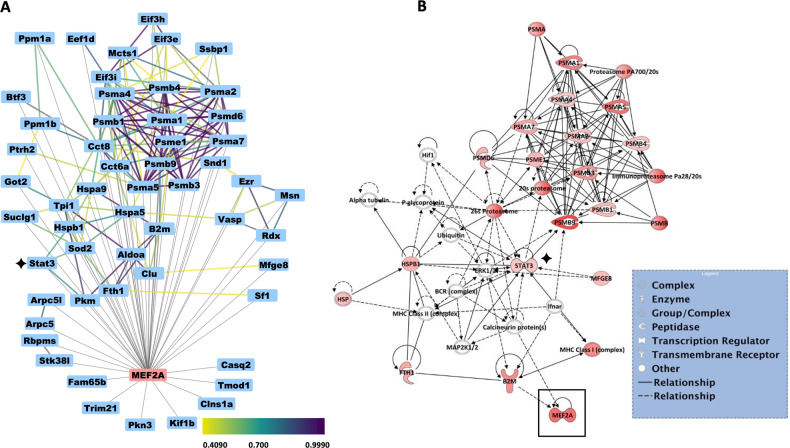
Protein networks associated with 56 enriched MEF2A interactors. **A** The network between identified proteins is built using the STRING database using confidence level of 0.4 (default setting) and with no additional interactors. The edge color corresponds to the confidence score depending on the database, as defined in the legend. The gray edges are added to the network and represent the novel protein-protein interaction network identified by our study. Node position and edge length and width are created arbitrarily. **B** The top scoring IPA protein networks ‘Gene expression, RNA Damage and Repair, RNA Post-Transcriptional Modification’ is depicted for the highly enriched proteins in MEF2A complex in PCMs. The shape of the node represents the molecular type of the proteins as defined in the legend. The red color intensity represents the magnitude of enrichment of the protein in the MEF2A complex. The protein interaction networks were generated with IPA (QIAGEN Inc., https://www.qiagenbioinformatics.com/products/ingenuity-pathway-analysis/). STAT3 gene is marked by a black star in both networks.

To further illuminate the biological and molecular interactions in the MEF2A interactome, we next built a network of protein-protein interactions using the STRING database using the Cytoscape Software platform (Fig. 2A). In the network, PSMA1, PSMA2, PSMA4, PSMA5, PSMB1, PSM4, PSM9, EIF3E, EIF3I, and EIF3H were found to interact with each other with a high confidence score. The network shows several novel interactions between the MEF2A gene and proteins such as STAT3, PKM, PKN3, ARPC5, RBPMS, STK38L, and TRIM21 in the PCMs. Of particular interest was the identified association with STAT3 which has a previously well-documented cardioprotective role in the heart [48–51]. In addition, the robust interaction with several actin binding proteins was also noted.

To further interrogate the data set, the 56 enriched proteins that met our cutoff criteria were analyzed using Qiagen’s Ingenuity Pathway Analysis software (IPA; www.ingenuity.com). Figure 2B indicates the top enriched network with the highest percentage of focus molecules. The top network was generated with the major function of Gene Expression, RNA damage and repair, RNA post-transcriptional modification. Notably, this network, along with the GO network depicted in Fig. 2A contains two transcription factors, STAT3 and MEF2A, that have previously been shown to independently play prominent roles in cardiomyocyte biology. Based on the IPA database, STAT3 protein is interconnected with different complexes such as Calcineurin, BCR, ERK1/2, MAP2K1/2, ubiquitin, and 26 s and 20 s proteasome complexes. Additionally, the network reveals that the Calcineurin complex is implicated as a common regulator between STAT3 and MEF2A.

### Biochemical validation of proteomic interactome data

**Fig. 3:**
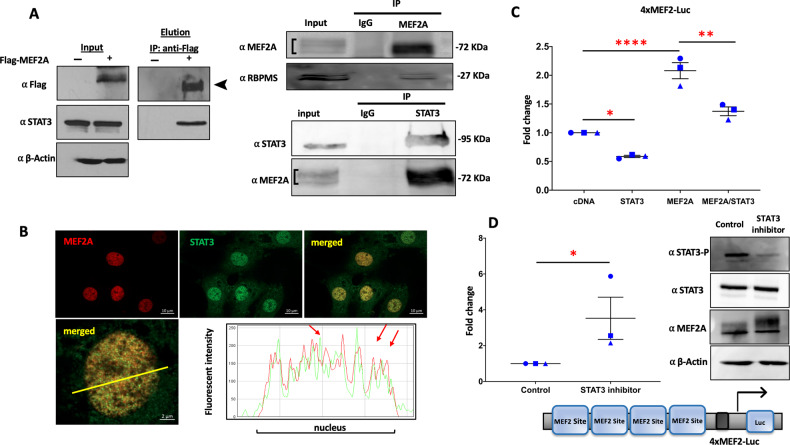
Validation of selected putative interactions. **A** PCMs were transfected with a Flag-MEF2A construct and lysates were assessed for expression by Western blotting. Flag-MEF2A lysates were used for IP using α-Flag magnetic beads and the eluates were blotted with anti-Flag and STAT3 antibodies. IP with lysates from non-transfected PCMs were used as negative controls (Left panel). Non-transfected PCM lysates were used for IP using MEF2A antibody to confirm the interaction between endogenous MEF2A and RBPMS. IP with IgG was used as controls (Top right). Non-transfected PCM lysates were used for IP using a STAT3 antibody IP to confirm the interaction between endogenous STAT3 and MEF2A. IP with IgG was used as control (Bottom right). Number of biological replicates carried out for the Western blot and IP data is *n* = 3. **B** Confocal immunofluorescence analysis of endogenous MEF2A and STAT3 indicates a nuclear localization in PCMs. PCMs were fixed and stained for MEF2A in red and counterstained for STAT3 in green. Slides were analyzed by confocal microscopy. The scale bars are 2 and 10 μm. The intensity blot of MEF2A and STAT3 signals is shown over the region shown by the yellow line in the merged image. **C** STAT3 and MEF2A expressing vectors alone and in combination were ectopically expressed in PCMs along with a 4xMEF2 luciferase reporter gene. Renilla luciferase was used to normalize for transfection efficiency. Lysates were collected at 48 h. The luciferase activity under each condition was measured and normalized to Renilla values to determine the fold change. **D** PCMs were transiently transfected with 4xMEF2 luciferase reporter gene for 48 h and then treated with STAT3 inhibitor (C188-9;10 µM) for 1 h in serum free medium. The control cells were treated by the solvent (DMSO) in serum free medium. Representative western blots of three independent biological replicates are presented which were carried out to assess the STAT3 inhibitor effect as compared to control condition. A schematic of the 4xMEF2A-Luc construct is shown below the luciferase data. Each condition in the luciferase reporter assay is compared to the respective control condition for three independently transfected samples (technical replicates) to determine the fold change. Each dot represents one biological replicate and corresponds to the mean of three technical replicates. The error bars represent standard error of the mean (SEM). Tukey’s multiple comparisons test in one-way ANOVA and independent two sample *t*-test using GraphPad Prism 8.0 were used to test for statistical significance. Adjusted *p*-value *≤0.05, **≤0.01, ***≤0.001****<0.0001, comparing to control.

To experimentally further validate the quantitative proteomics data, a biochemical IP approach was employed. To this end, we tested the MEF2A interaction with a subset of its candidate interactors (RBPMS and STAT3). Lysates from Flag-MEF2A expressing cardiomyocytes were used for IP using α-Flag magnetic bead-based IP, with lysates from control primary cells serving as negative control in these experiments. Western blotting of the respective eluate fractions revealed that STAT3 co-immunoprecipitated with MEF2A (Fig. 3A), whereas there was no detection of STAT3 in control IP eluate fractions. Subsequently, we tested whether endogenous interactions between MEF2A and RBPMS or STAT3 could also be detected. Indeed, Western blot analyses of endogenous MEF2A co-immunoprecipitated eluates validated the co-enrichment of RBPMS proteins (Fig. 3A top). Similarly, Western blot analyses of reciprocal STAT3 immunoprecipitate eluates also resulted in the detection of endogenous MEF2A (Fig. 3A bottom), confirming an interaction between endogenous MEF2A and STAT3. To understand whether the MEF2A/STAT3 complex persists during cardiomyocyte remodelling, an IP experiment was performed after Flag-MEF2A was expressed in PCMs followed by treatment with Isoproterenol (ISO) for 24 h which induces PCM remodelling events. This analysis confirmed the persistence of the STAT3 in MEF2A complex in ISO-treated PCMs (Supplementary Fig. S1).

Since STAT3 is a nuclear transcription factor, we next aimed to ascertain whether it co-localizes with MEF2A protein in PCMs using immunofluorescence analyses. Figure 3B documents the subcellular localization of STAT3 and MEF2A [52] in PCMs. Immunofluorescence analyses indicated a higher level of STAT3 and MEF2A in the nucleus, relative to the cytoplasm, although there is evidence of low levels of STAT3 present in both compartments. Line scan analyses of these micrographs indicate that, while there are STAT3 and MEF2A puncta that do not co-localize, there are also several co-localized peaks, consistent with the interpretation that a subset of STAT3 and MEF2A proteins are co-localized in the nucleus and exhibit overlapping distributions (Fig. 3B).

### Function of STAT3/MEF2A protein:protein interaction in PCMs

To further investigate the nature of the MEF2A:STAT3 interaction, we assessed the functional effect of STAT3 on MEF2A transactivation properties using a MEF2-dependent luciferase reporter gene assay (Fig. 3C). STAT3 and MEF2A were exogenously expressed alone or in combination in PCMs. These data indicate that ectopic MEF2A expression activates MEF2-luc over the background MEF2 activity in these cells. We have observed previously that there is a substantial activation of the reporter by endogenous MEF2 in this system. Ectopic expression of STAT3 in this assay resulted in inhibition of MEF2A-mediated transcriptional activation. This inhibition was apparent on both the endogenous activation of the reporter gene when comparing lanes 1 and 2 and also the transfected MEF2A in lanes 3 and 4 (Fig. 3C).

Since STAT3 phosphorylation at Y705 was reported previously to increase STAT3 dimerization and translocation to the nucleus, DNA binding and stimulation of gene expression [53], we subsequently tested if the MEF2 interaction might be modified by STAT3 post-translational modification. STAT3 phosphorylation is mediated by the Janus Kinase (JAK) family, including epidermal growth factor and Src [54], and this regulation can be assessed using an inhibitor of STAT3 phosphorylation (STAT3 inhibitor XIII, C188-9). Using this inhibitor based approach in PCMs, Western blotting confirmed that STAT3 Y705 phosphorylation was indeed reduced by the inhibitor treatment in cultured PCMs. Under these conditions, we also observed an increase in MEF2-dependent luciferase activation consistent with a repressive effect of STAT3 on MEF2 transactivation properties (Fig. 3D).

**Fig. 4:**
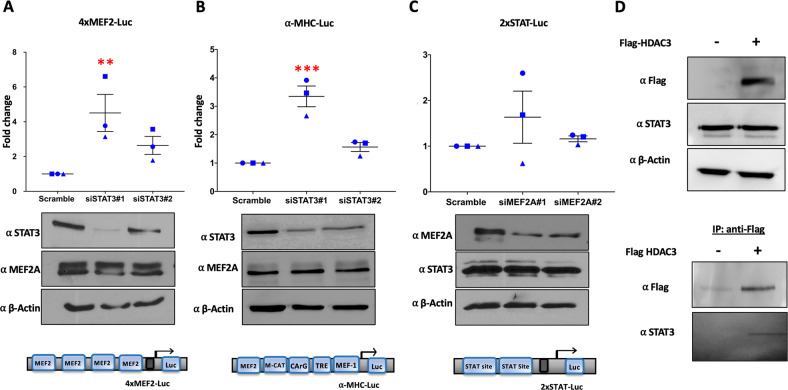
STAT3 regulates MEF2A-dependent transcription. **A** PCMs were transfected with a 4xMEF2 luciferase reporter gene along with two independent siRNAs, siSTAT3#1 and #2, to deplete the endogenous STAT3 levels or a scrambled siRNA was used as control. Renilla luciferase was used to normalize for transfection efficiency. Lysates were collected at 48 h. The luciferase activity under each condition was measured and normalized to Renilla values to determine the fold change (Top panel). Corresponding western blot analysis using equal amounts of total protein of the cell lysates were used to confirm siRNA mediated STAT3 depletion as compared to scrambled controls (Bottom panel). Number of biological replicates for western blotting was *n* = 3. A schematic of 4xMEF2A-Luc construct is shown below the luciferase results. **B** PCMs were transfected with an α-MHC-Luc reporter gene with two independent siRNAs, siSTAT3#1 and #2, or scrambled siRNA as control. Luciferase activity was assessed after 48 h and normalized to Renilla activity (Top panel). Corresponding western blot analysis of cell lysate to confirm the depletion of STAT3 protein level as compared to the control is shown (Bottom panel). Number of biological replicates for this analysis was *n* = 3. Schematic of α-MHC-Luc construct is shown below the luciferase data. **C** PCMs were transiently transfected with 2xSTAT-Luc reporter gene with two independent siRNAs, siMEF2A#1 and #2, or scrambled siRNA as control. Luciferase activity was assessed after 48 h and normalized to Renilla activity (Top panel). Corresponding western blot analysis of cell lysate to confirm the depletion of MEF2A protein level as compared to the control (Bottom panel). Number of biological replicates carried out for western blotting was *n* = 3. A schematic of the 2xSTAT-Luc construct used is shown below the luciferase data. **D** PCMs were transiently transfected with a Flag-HDAC3 construct and lysates were assessed for protein expression by Western blotting. Flag-MEF2A lysate was used for IP using α-Flag magnetic beads and the eluates were blotted with anti-Flag and STAT3 antibodies. IP with lysate from non-transfected PCMs were used as negative controls. Number of biological replicates for western blot and IP carried out was *n* = 3. Each condition in the luciferase reporter assay is compared to the control condition for three independently transfected samples (technical replicates) to determine the fold change. Each dot represents one biological replicate and corresponds to the mean of three technical replicates. The error bars represent standard error of the mean (SEM). Dunnett’s multiple comparisons test in one-way analysis of variance using GraphPad Prism 8.0 was used to test for statistical significance. Adjusted *p*-value *≤0.05, **≤0.01, ***≤0.001****<0.0001, comparing to control.

Next, we employed a loss of function analysis using siRNA targeting of STAT3 in PCMs. For that, we utilized reporter gene assays in which PCMs were transfected by 2 independent siRNAs targeting STAT3 or a scrambled control along with 4xMEF2-luc as a reporter of MEF2 activity (Fig. 4). Western blot analysis confirmed a considerable reduction in protein levels of STAT3 using siSTAT3#1 and #2 as compared to scrambled controls (Fig. 4A). The reporter gene data (Fig. 4A top panel) indicate that STAT3 depletion resulted in an increase in MEF2 reporter gene activation further substantiating the repressive effect of STAT3 on MEF2A function.

Since these initial assays were done using synthetic reporter genes, we next utilized a natural rather than synthetic promoter region to assess this interaction. The regulatory region (−340 to +20) of the α-myosin heavy chain (α-MHC) gene has been extensively studied and it has been confirmed that this sequence is sufficient for directing cardiac specific expression [28]. Within this region, the MEF2 binding site is located between −314 and −327 of the regulatory region proximal to the transcription start site [55]. Therefore, we utilized a reporter gene assay in PCMs in which the α-MHC promoter drives a luciferase reporter gene. Using this reporter gene, the data indicate that STAT3 depletion increases the activity of α-MHC-luc activity (Fig. 4B). Again, western analysis confirmed the reduction of STAT3 levels using siRNA as compared to the scrambled controls (Fig. 4B). Collectively, these experimental approaches of gain of function (ectopic expression), loss of function (siRNA depletion) and pharmacologic inhibition (STAT3 inhibitor XIII, C188-9) on synthetic and natural MEF2 dependent promoter regions collectively indicate that STAT3 represses MEF2 transactivation function. It should be noted however that the specific function of the MEF2A/STAT3 interaction is complex and could well vary depending on the unique architecture of each promoter region. The dynamic regulation of each promoter/enhancer region therefore requires that, in all likelihood, each gene regulatory region needs to be assessed independently in terms of its regulation by the MEF2A/STAT3 interaction since the impact will depend on the combinatorial nature of the promoter/enhancer landscape.

We also addressed the question whether MEF2A might have a corresponding effect on STAT3 function through its cognate binding sites. To study this, we used a synthetic reporter gene containing two STAT3 binding sites driving a luciferase reporter gene in PCMs. Unlike the data clearly indicating the repressive effect of STAT3 on MEF2 function, we observed that MEF2A depletion may have a slight but not easily discernible and reproducible effect on the STAT-luc reporter gene activation (Fig. 4C), indicating that the repressive effect of STAT3 on MEF2A function seems more robust than the reciprocal effect of MEF2A on STAT3 function. This should be qualified by the recognition that this is observed on a synthetic STAT3 dependent reporter gene. As alluded to above, the full impact of the MEF2A/STAT3 protein interaction may be much more dynamic on a genome wide level. This idea is supported by further experimentation that we undertook (described below) in which depletion of either factor can have fundamental effects on cardiomyocyte responsiveness to a hypertrophic signal (Phenylephrine treatment).

Since STAT3 has previously been shown to recruit HDAC3 we postulated that HDAC3 might be involved in the repressive effect of STAT3 on MEF2A function. Moreover, STAT3 is also known to be a non-histone substrate for HDAC3 in different cell types [56, 57]. As is well documented, HDACs do exist in large multi-subunit complexes that have crucial functions in chromatin modification and transcription modification [58]. We therefore tested the possibility that HDAC3 may associate with STAT3 in cardiomyocytes. The data depicted in the IP analysis shown in Fig. 4D does indeed support the interaction between STAT3 and HDAC3 in PCMs. Of note, previous studies revealed that HDAC3 regulates STAT3 in B-cell lymphoma by affecting the STAT3 acetylation level and in turn STAT3 nuclear export [59]. To address this possibility in PCMs, the effect of HDAC3 and STAT3 inhibitors on STAT3 localization in PCMs was carried out. PCMs were treated with an HDAC3 inhibitor (RGFP966) or STAT3 inhibitor (STAT3 inhibitor XIII, C188-9) and immuno-stained with MEF2A and STAT3 antibodies (Supplementary Fig. S2). In the control condition, STAT3 was detected mostly in the nuclear compartment; however, with both HDAC3 and STAT3 inhibitors, the distribution of STAT3 was more diffuse in both the cytoplasm and nucleus and considerably less signal was detected in the nucleus. Further, we assessed the ability of HDAC3 to affect the binding of STAT3 and MEF2A. For this, Flag-MEF2A was expressed in PCMs which were then treated with an HDAC3 inhibitor (RGFP966) or STAT3 inhibitor (STAT3 inhibitor XIII, (C188-9). Consistent with previously published data [59], HDAC3 inhibition decreased STAT3 phosphorylation similar to the effect of the STAT3 inhibitor (Supplementary Fig. S3). Bound proteins were precipitated using the Flag tag and immunoblotted with anti-MEF2A and anti-STAT3. Supplementary Fig. S3 indicates that STAT3 co-precipitated with Flag-MEF2A, indicating that they can still interact under conditions of HDAC3 or STAT3 inhibition.

### Transcriptome profiling of STAT3 depleted PCMs

**Fig. 5:**
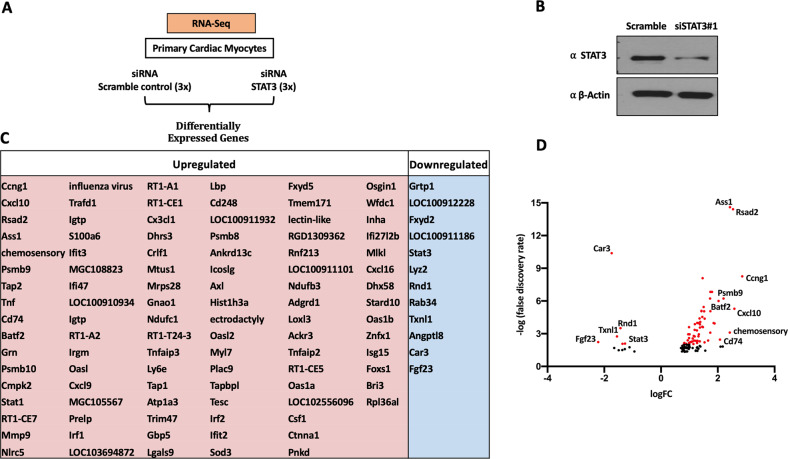
Identification of differential expressed genes in STAT3 knock down in PCMs. **A** Workflow of RNA sequencing in PCMs. PCMs were transiently transfected by siRNA targeting STAT3 (siSTAT3#1) to deplete the endogenous STAT3 level or a scrambled siRNA used as controls at 48 h. RNAs were extracted for next gen-sequencing. **B** PCMs were transiently transfected with siSTAT3#1 or scrambled siRNA as control. Lysates were collecting at 48 h. Equal amounts of proteins were assessed by western blotting analysis to confirm siRNA mediated STAT3 depletion as compared to scrambled controls. Data are representative of *n* = 3 biological replicates. **C** Genes identified by RNA-seq analysis that are upregulated (Red) or downregulated (Blue) in STAT3 depleted PCMs (FDR < 0.05). **D** A volcano plot indicates upregulated and downregulated genes in siSTAT3 RNA-seq data. Black data point if FDR value >0.01. Red data points if FDR value < 0.01. Labels of the genes are shown if −1.35 > logFC > 2 and FDR value <0.01.

Previously, we have documented transcriptome changes in MEF2A depleted PCMs using RNA-seq to assess the transcriptome [60]. Based on the success of this approach in identifying important genes and cellular processes regulated by MEF2A, we utilized the same approach in analyzing STAT3. We reasoned that, apart from revealing more about the function of STAT3 in PCMs, it would also allow us to compare both data sets since they were performed under identical conditions, to potentially identify co-regulated genes. We initially hypothesized that, in view of the protein interaction between STAT3 and MEF2A, that they might share a number of common target genes. Therefore, we used STAT3 siRNA depletion coupled with transcriptome analysis (RNA-seq) to address this question. Depletion of STAT3 by siRNA technology in PCMs was carried out and RNA from the samples was prepared for RNA-seq analysis (Fig. 5A). The depletion of STAT3 was confirmed by western blotting before sequencing (Fig. 5B). The RNA-seq data from this experiment documented 111 differentially expressed genes (DEGs), of which 99 were upregulated and 12 were downregulated with siRNA STAT3 compared to the scrambled control in cardiomyocytes (threshold FDR ≤ 0.05) (Fig. 5C). This observation is consistent with a mostly repressive effect of STAT3 in PCMs. Additionally, a volcano plot was generated demonstrating the log2 Fold Change of the differentially expressed genes (Fig. 5D). Firstly, the data indicates that STAT3 was significantly downregulated with FDR value <0.01 and log FC < −1.35 in the siRNA depleted cardiomyocytes compared to the scrambled control, indicating the efficacy of the approach. This analysis illustrates that STAT3 downregulation was accompanied by downregulation of some genes such as Fibroblast Growth Factor 23 (FGF23), Carbonic Anhydrase III, Muscle Specific (CAR3), and Rho Family GTPase 1 (RND1), while a large number of genes such as Cyclin G1(CCNG1), C-X-C Motif Chemokine Ligand 10 (CXCL10), and CD74 Molecule (CD74) were upregulated. These whole PCM transcriptome data support our more targeted biochemical assays indicating that STAT3 can function as both a transcriptional activator as well as a repressor, and that its repressor function seems to be somewhat prevalent in PCMs.

To understand more about the differentially regulated genes in the STAT3 depleted PCMs, bioinformatic analysis of the 111 DEGs was carried out using the David 6.8 functional annotation tool as depicted in supplementary document 2. We used a functional annotation clustering tool to group relevant annotations and reduce the redundancy between them. The higher Enrichment scores are accompanied by a lower p-value of annotation terms (*p*-value < 0.05). The top clusters with the highest scores are identified in supplementary document 2. Functional annotation revealed that the STAT3-regulated genes were enriched with genes involved in the defense response, innate immune response, and regulation of the adaptive immune response. Cluster 1 was enriched in genes involved in defense mechanisms and response to cytokines, consistent with the previously documented cardioprotective role of STAT3. Cluster 2 grouped genes are mainly related to the innate immune response, response to biotic stimulus and defense response to virus. In Cluster 2, genes such as CXCL9, TNFAIP3, TNF, IRF1, IFIT2, IFIT3, CXCL16, CXCL10, and MMP9 were upregulated in STAT3 depleted cardiomyocytes. Interestingly, clusters 3, 4, 5, 6, and 7 were all related to the cellular response to lipopolysaccharide, antigen processing and presentation, and antiviral defense. Cluster 8 was enriched with genes related to the regulation of leukocyte-mediated cytotoxicity and regulation of the adaptive immune response, such as RSAD2, TAP1, RT1-A1, and RT1-A2 genes. Thus, at the mRNA level, STAT3 seems to play a prevalent role in regulating genes involved in immune and inflammatory responses. An observation consistent with its function as a ‘protective’ factor in cardiomyocyte biology.

### Comparative transcriptome profiling of STAT3 and MEF2A transcription factors in cardiomyocytes

**Fig. 6:**
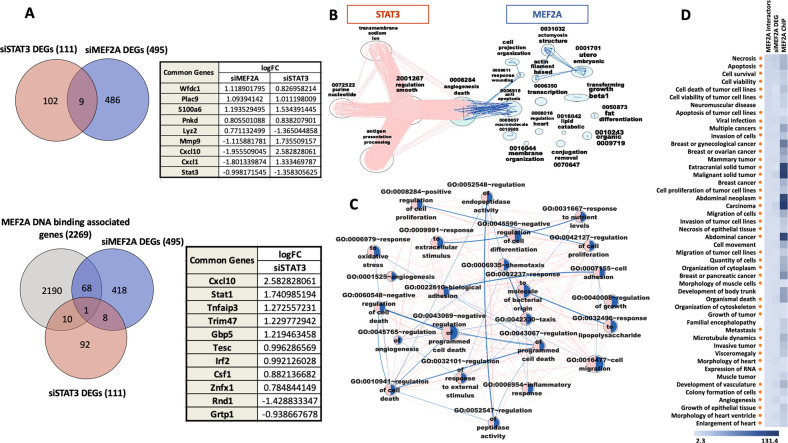
Comparison between siMEF2A and siSTAT3 datasets. **A** 9 genes are common between DEGs in siMEF2A and siSTAT3 datasets in PCMs that are indicated in the Venn diagram (Top left). Table represents logFC of the 9 common genes in response to MEF2A and STAT3 depletion in PCMs (Top right). A Venn diagram represents the common genes between the three data sets: MEF2A DNA binding associated genes, siMEF2A DEGs, and siSTAT3 DEGs (Bottom left). Table represents logFC of the 11 common genes between MEF2A DNA binding associated genes and siSTAT3 DEGs (Bottom right). **B** The enriched GO BP terms for each dataset have been clustered using an Autoannotation plugin in Cytoscape software. The blue clusters identify the specific processes related to MEF2A in PCMs. The red clusters indicate the specific processes related to STAT3 in cardiac cells. The green cluster shows the common processes between MEF2A and STAT3 in primary cardiomyocytes. The edges between clusters show the shared genes between clusters. The cluster size and position are created arbitrarily. **C** 24 GO BP terms are common between siMEF2A and siSTAT3 datasets in PCMs. The node size represents the gene size. The edge represents the common genes between the terms. Node position and edge length are created arbitrarily. **D** Integrative analysis of omics data using proteomics, transcriptomics, and ChIP-exo experiments reveals multi-functions of MEF2A in myocardial cells. Three types of omics data have been studied to depict MEF2A gene function in cardiac cells. A Heat map is generated using the three omics technologies and the IPA database used to identify Diseases and Biofunctions that are common in the three omics data. Heatmap indicates the score represented in -log(*p*-value) for the identified 48 Diseases and Biofunctions. The red dots beside the map indicate possible involvement of STAT3 in the MEF2A complex.

Our group has previously studied transcriptome analysis in MEF2A depleted PCMs [60]. Gene Ontology analysis of the DEGs showed enrichment of GO Biological Processes that are related to cell death and the inflammatory response in PCMs [60]. Subsequently, a functional pro-survival role of MEF2A in PCMs was characterized by us [61]. Since the current study has revealed an interaction between MEF2A and STAT3, we undertook a comparative analysis of the transcriptomes of MEF2A and STAT3 siRNA depleted cardiomyocytes to assess the potential overlap of gene regulatory programs or mechanisms shared between the two proteins. By comparing the identified DEGs in STAT3 (111 RNAs) and MEF2A (495 RNAs) in PCMs, we observed 9 DEGs in common between the 2 data sets (Fig. 6A). The corresponding genes are listed in the table which indicates the gene expression changes in both data sets. In view of the functional data that we had acquired on the interaction of MEF2A and STAT3 and the effects of MEF2A or STAT3 depletion on the PCM transcriptome, we were particularly interested in genes that were both upregulated by STAT3 depletion and down regulated by MEF2A depletion. Genes exhibiting this pattern of expression are: lysozyme 2 (LYZ2), C-X3-C Motif Chemokine Ligand 1 (CX3CL1), C-X-C Motif Chemokine Ligand 10 (CXCL10), and Matrix Metallopeptidase 9 (MMP9).

We further interrogated these data by co-analyzing MEF2A DNA binding associated genes in PCMs that were identified previously by ChIP-exo analysis in our lab [62]. This analysis revealed an interesting observation that approximately 10% of the STAT3 depletion associated DEGs are concomitantly bound by MEF2A as indicated by ChIP-exo. Paradoxically, these gene products were not seen to change in MEF2 depleted CMs in transcriptome analysis. These observations indicate a complex and possibly partially redundant relationship between these factors that is difficult to tease apart by monogenic depletion analysis (see “Discussion”). The analysis shows that there are 69 genes in common between ChIP-exo and siMEF2A RNA-seq experiments (Fig. 6A bottom and Supplementary Document 3). Based on this analysis, these genes have been differentially expressed in MEF2A depleted conditions and also associated with MEF2A enrichment peaks in ChIP analysis. Moreover, this analysis identified 10 genes in common between MEF2A ChIP-exo data and siRNA STAT3 RNA-seq experiments. These 10 genes are listed in the lower table in Fig. 6A.

GO Biological Processes related to siSTAT3 DEGs were compared to GO BP previously identified with siMEF2A DEGs (Fig. 6B). The GO analysis identifies 24 Biological Process terms shared between siMEF2A and siSTAT3 DEG lists in PCMs such as GO:0010941 “regulation of cell death”, GO:0043069 “negative regulation of programmed cell death”, GO:0060548 “negative regulation of cell death”, GO:0006954 “inflammatory response”, GO:0006979 “response to oxidative stress”, and GO:0045765 “regulation of angiogenesis” (Fig. 6C). While there is much to learn about the complex genetic programs and redundancy mediated by MEF2A and STAT3 proteins individually and as a complex, it appears that they preside over critical gene regulatory networks in cardiomyocytes.

### Regulation of cardiomyocyte MMP9 expression by MEF2A and STAT3

**Fig. 7:**
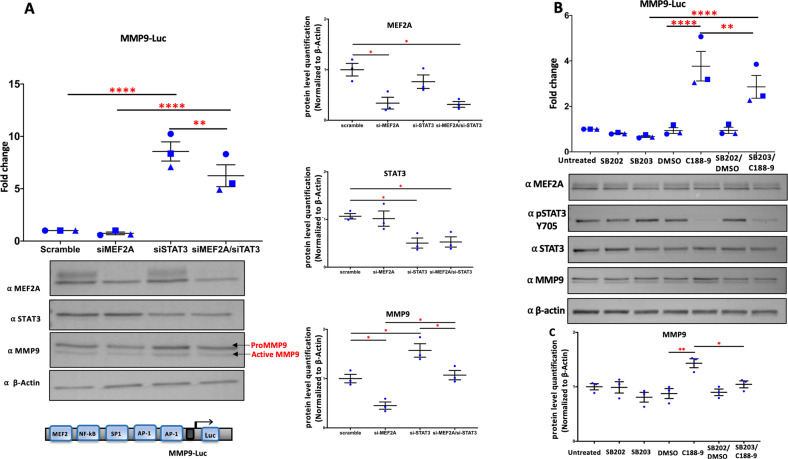
MEF2A/STAT3 complex regulates the MMP9 expression in cardiomyocytes. **A** PCMs were transiently transfected with an MMP9 luciferase reporter gene along with siRNA targeting the endogenous MEF2A and STAT3 levels individually and in combination. Scrambled siRNA was used as a control. Renilla luciferase was used to normalize for transfection efficiency. Lysates were collected at 48 h. The luciferase activity under each condition was measured and normalized to Renilla values to determine the fold change. *N* = 3 biological replicates per condition (Top panel). A representative western blot for three biological replicates for MEF2A, STAT3, MMP9, and β-actin is presented. Corresponding western blot analysis was used to confirm siRNA mediated MEF2A and STAT3 depletion as compared to scrambled controls (Bottom panel). A schematic of the MMP9-Luc construct is shown below the representative western blot. The expression of MEF2A, STAT3, and MMP9 are assessed using western blot analysis. The dot graphs represent the level of MEF2A, STAT3, and MMP9 proteins after normalization to β-actin (right panel). Both prominent bands (hyper and hypophosphrylated) for MEF2A are included together in the quantification. Data are presented as mean ± SEM. *n* = 3 *P < 0.05 vs control. **B** PCMs were transfected with the MMP9 luciferase reporter gene and harvested at 48 h. On day1 after transfection, cells were treated with the p38 MAPK inhibitor (SB203580; 10 μM) or its inactive analogue (SB202474; 10 μM) for 24 h in a serum-free medium. Cells were treated with a STAT3 inhibitor (C188-9;10 µM) as indicated, and the control cells were treated with the corresponding solvent (DMSO). Luciferase values were normalized to Renilla. *N* = 3 biological replicates per condition. Representative western blot for three biological replicates for MEF2A, pSTAT3, STAT3, MMP9, and β-actin. Corresponding western blot analysis of the cell lysates was used to confirm SB203580 and C188-9 treatments as compared to controls as indicated (Bottom Panel). **C** The plot represents the MMP9 protein levels after normalization to β-actin. Each condition in the luciferase reporter is compared to the respective control condition for three independently transfected samples (technical replicates) to determine the fold change. Each dot represents one biological replicate and corresponds to the mean of three technical replicates. The error bars represent standard error of the mean (SEM). Tukey’s multiple comparisons test in one-way ANOVA using GraphPad Prism 8.0 were used to test for statistical significance. Adjusted *p*-value *≤0.05, **≤0.01, ***≤0.001, ****<0.0001, comparing to control.

Next, we took a more targeted approach to analyze the MMP9 gene since it was identified as being potently regulated by both MEF2A and STAT3 in the PCM transcriptome analysis. In Fig. 7A we document that STAT3 depletion promotes a corresponding increase in MMP9 protein level which is consistent with the transcriptome data. MEF2A depletion had a marginal effect in this assay that was again consistent with the transcriptome data. Figure 7B depicts experiments in which we manipulated STAT3 activity using a STAT3 tyrosine 705 inhibitor (C-188-9) or a p38 MAP kinase inhibitor (SB203580) that reduces MEF2A activity, using an MMP9 luc assay as the readout. Again, we observed a potent effect of STAT3 repression in hyperactivating the MMP9 promoter and a more marginal effect of p38 MAP kinase inhibition (which targets MEF2A). The reporter gene data is mirrored in protein level analysis of MMP9 in Fig. 7C.

### Effects of MEF2A/STAT3 depletion on phenylephrine induced PCM hypertrophy

**Fig. 8:**
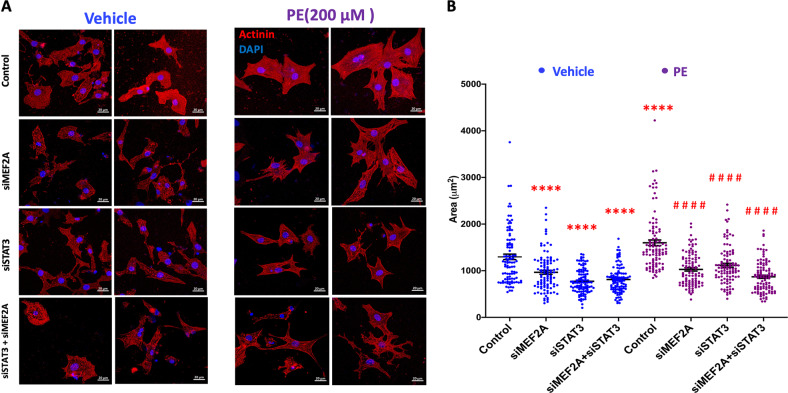
MEF2A/STAT3 complex is involved in biological processes in myocardial cells. **A** MEF2A and STAT3 are involved in the increase of cardiomyocyte surface area in response to phenylephrine. Immunofluorescence analysis of α-Actinin and DAPI was performed in PCMs transfected with siRNA targeting MEF2A and STAT3 individually and in combination for 48 h then treated with PE (200 μM) or vehicle (H_2_O) for a further 48 h. The figure illustrates 2 representative images for each condition. The scale bar is 20 μm. **B** The scatter plot represents the quantification of the surface area of α-Actinin-positive cells using Image J software. The blue dots represent PCMs treated with H_2_O and purple dots represent the PCMs treated with PE. The data presented are derived from the surface areas of 100 cells that were randomly selected from four biological replicates (25 cells per biological replicate). Each cell surface area measurement is represented as a dot in each condition and the mean is indicated by a horizontal line. Tukey’s Multiple comparisons test in one-way ANOVA using GraphPad Prism 8.0 was used to test for statistical significance. * represents statistical significance from control vehicle-treated group. # represents statistical significance from PE-treated group. Adjusted *p*-value *≤0.05, **≤0.01, ***≤0.001, ****<0.0001, comparing to control.

It has previously been documented in many studies that phenylephrine (PE) treatment induces neonatal rat PCM hypertrophy in vitro. We took advantage of this well characterized model of cardiac hypertrophy to assess the role of MEF2A and STAT3 in this cellular process since both proteins have been previously independently implicated in cardiac hypertrophy [2, 15, 63, 64]. We documented that PE treatment does indeed induce an increase in PCM surface area consistent with previous reports (Fig. 8A, B). Moreover, depletion of MEF2A, STAT3 or both together significantly blunted the hypertrophic response with the most potent effect being seen with the compound depletion of both proteins together (Fig. 8B).

## Discussion

The MEF2A transcriptional regulator plays a pivotal role in cardiomyocyte differentiation and in response to stress signaling in the heart [13, 65]. Additionally, MEF2A has differential regulatory effects on a panoply of genes in both the atria and ventricles of the adult heart [62, 66]. MEF2 has also been strongly implicated in aberrant patterns of cardiomyocyte gene expression in the adult heart leading to cardiac hypertrophy and heart failure. The versatility of MEF2A’s roles in the heart and other body systems has so far revealed that post-translational modifications (PTMs) and complex tissue specific protein-protein interactions likely subserve its diverse functions. Here, we document the MEF2A interactome in primary rat cardiomyocytes using quantitative mass spectrometry (iTRAQ-LC-MS/MS) analysis. Our interactome analysis reveals a unique set of novel protein-protein interaction that may play important roles in the control of cardiomyocyte gene expression. Of particular interest are MEF2A interactions with STAT3, PKM2, PKN3, and RBPMS in view of their previously documented prominent roles in the heart. Collectively, this novel cardiomyocyte interactome will allow a more thorough understanding of MEF2A function in gene networks in the heart and possibly lead to important biological insights regarding the role of MEF2A in the heart under pathologic conditions such as cardiac hypertrophy and heart failure.

Since one of the prominent interactions with MEF2A in our data set is STAT3, it is intriguing that STAT3 has a reported cardioprotective role in several pathological conditions in the heart by improving survival [48], decreasing oxidative stress [49, 50, 67] increasing angiogenesis [48, 51], and regulating metabolism [68]. The pro-survival role of STAT3 is reported to occur through inhibition of apoptosis in the ischemic myocardium. Activation of granulocyte colony-stimulating factor receptor (G-CSFR) by the JAK/STAT3 pathway improves cardiac function by upregulation of anti-apoptotic molecules, Bcl-2 and Bcl-xL [48]. Moreover, it is also documented that the gp130/STAT3 pathway is essential for cardiomyocyte hypertrophy in pressure-overload hypertrophy model mice [63, 64, 69]. Similar to STAT3, MEF2A has also been implicated in regulating PCM cell death and hypertrophic responses by ourselves and others [2, 15, 61].

PKM2, like MEF2A, is expressed in cardiac cells during development and immediately after birth but sparingly during adulthood [70]. Also, PKM2 is highly expressed in failing human hearts compared to non-failing hearts [71]. Another kinase, protein kinase N3 (PKN3), is ubiquitously expressed in human tissues with distinct roles in the cytoplasm and nucleus. More specifically, PKN3 has been implicated in transcriptional regulation of the ANP gene that is associated with cardiac hypertrophy in response to phenylephrine [72]. The ANP gene is also regulated by MEF2A [73]. Another GO process of interest with regard to MEF2A protein-protein interactions is regulation of alternative splicing since there is substantial evidence of co-transcriptional splicing that might implicate MEF2A in recruiting splicing factors to actively transcribed genes. In support of this, proteins of the RBPMS family were identified in the MEF2 interactome. RBPMS and RBPMS2 proteins have been shown to regulate alternative splicing that promotes sarcomeric organization and calcium handling [74]. Thus, a number of splicing-related factors are associated with MEF2A suggesting the possibility that it might serve to co-ordinate co-transcriptional splicing.

### Network analysis of MEF2 interactome components

Our group has utilized high-throughput analysis at different molecular levels to investigate the role of the MEF2A gene in cardiac muscle cells [60, 61]. The role of the MEF2A gene has been documented by transcriptome analysis by determining DEGs in response to MEF2A depletion. Additionally, chromatin-based genome-wide analysis of MEF2A DNA binding has identified MEF2A target genes in cardiac muscle with divergent functions. Indeed, biological function involves the interactive system of molecular entities i.e., DNA, RNA, proteins, and metabolites that are highly linked to physiological states associated with disease processes. Hence, the integration of different types of omics data, termed multiomics, may lead to fundamental multifactorial insights into complex biological systems allowing inference of causal relationships implicated in cellular function. Here, we have begun to assess the role of MEF2A using an integrated multiomic approach. Our analysis of three distinct omics data sets has allowed identification of cellular processes that MEF2A may serve to regulate in the heart including necrosis, apoptosis, response to viral infection and enlargement of the heart. Also, these data highlight the involvement of STAT3 as a modulator of MEF2A function in this context.

Interestingly, some actin-binding proteins have been identified in our proteomic analysis, such as actin-related protein 2/3 complex subunit 5 (ARPC5), actin-related protein 2/3 complex subunit 5-like protein (ARPC5L), Ezrin, Radixin, Moesin, and VASP. ARPC5 and ARPC5L are components of the ARP2/3 complex that play an important role in actin nucleation and formation of a branched actin-cytoskeleton structure [75–79]. The function of the ARP2/3 complex is essential for cell migration and protrusion formation. Additionally, the ARP2/3 complex plays a central role in signalling-induced actin-assembly [80, 81]. Besides the fundamental role of the ARP2/3 complex in the cytoplasm, the ARP2/3 complex is located in the nucleus and plays a crucial role in de novo polymerization in the nucleus [82–84]. Evidence suggests that the ARP2/3 complex is an actin polymerization regulator that was found associated with WASP [85] and cofilin [86], the actin disassembly factor, to regulate RNA-polymerase-II-transcription [87]. The ARP2/3 complex contributes to actin dynamics that regulate the transcriptional process and cytoskeletal homeostasis. In addition, Ezrin, Radixin, and Moesin, that we have identified as MEF2A interactors, are related proteins belonging to the ERM family that are conserved across species from worms to humans [88]. ERM proteins are known to be actin-binding proteins [89] and studies have documented the involvement of ERM proteins in numerous signal transduction pathways [90–92]. In terms of sub-cellular localization, ERM proteins have been found within the nucleus in different species [93–96], participating in different functions, such as mRNA export [97] and gene expression [98–100]. The potential roles of actin dynamics in cardiomyocyte functions and heart disease development have been recently reviewed [101]. Based on our findings, we speculate that MEF2A activity may be linked to actin dynamics in the heart.

### STAT3 association with MEF2A in cardiomyocytes

Our analysis reveals that STAT3 interacts with MEF2A both physically and functionally. We document an inhibitory role of STAT3 on MEF2A *trans*activation properties using STAT3 gain and loss of function approaches. These data indicate that the balance between the two transcription factors may be crucial in determining their functional specificity in the cardiac context. The therapeutic roles of STAT3 in heart disease have been reported in various studies demonstrating that STAT3 is cardioprotective in pathological conditions such as myocardial infarction, ischemia/reperfusion and doxorubicin-induced dysfunction [102, 103]. In cardiomyocyte-specific STAT3 knockout mice using α-MHC-driven Cre expression in conjunction with a loxP-site flanked exons 18, 19, and 20 of the murine STAT3 allele, STAT3-depletion in cardiomyocytes showed high rates of apoptosis and fibrosis and reduction in capillary density. It was suggested that these changes contribute to the significant alterations in the left ventricular systolic function [102]. Microarray analysis of cardiac tissue in these mice documented upregulation of genes associated with fibrosis and anti-angiogenesis. The mechanism underlying the role of STAT3 in cardiac muscle is poorly understood, and it has not been thoroughly explored at the level of the transcriptome. Here, we examined analyses of DEGs associated with STAT3 depletion. These data indicate a strong enrichment for genes associated with the defence response to cytokines, biotic and virus infection, and antigen representation. Indeed, myocarditis in humans, a result of injury and inflammatory processes, invokes stimulation of the immune system and genetic responses in the progression to heart failure [62, 104–107]. It is noteworthy that circulating levels of interleukin (IL)-6-related cytokines are predictors of mortality in human heart failure, while the expression and post-translational modifications of STAT3 are severely reduced in myocardial cells in patients with dilated cardiomyopathy [108]. In this context, STAT3 protein can act as a direct transcriptional inhibitor of inflammation. Thus, fibrosis, premature death, and dilated cardiomyopathy may be partially explained by the inflammatory response in STAT3 depleted cardiomyocytes. In this context, our findings support the previous idea that cardiomyocytes with STAT3 downregulation are more susceptible to pathogen-induced cardiac injury. Interestingly, in a previous study from our group, we identified a group of enriched genes that were differentially expressed during pressure overload-induced cardiac hypertrophy (TAC) which are responsible for positive regulation of the immune system including lymphocyte and leukocyte activation and proliferation [60], that was also reported in human heart failure and cardiomyopathy [109, 110]. We also demonstrated the function of MEF2A in suppressing genes associated with the inflammatory response in the heart [60]. An observation consistent with the requirement for balance between STAT3 and MEF2A in cardiomyocytes.

### Common and non-overlapping target genes of STAT3 and MEF2A: implications for heart function and disease

First, we examined common gene targets affected by STAT3 and MEF2A (previously performed in our lab). Common genes identified in the data sets, whose expression is repressed by STAT3 and enhanced by MEF2A, are CX3CL1, CXCL10 and MMP9. CX3CL1 is a cytokine that is upregulated in the myocardium of patients with chronic HF and the cardiomyocytes of mice with myocardial infarction (MI) [111]. CXCL10 is a known chemoattractant for T cell infiltration and a polarizing factor for the proinflammatory phenotype [112]. Of note, the CXCL10 level is elevated in cardiovascular disease [112]. MMP9 is secreted by cardiomyocytes [113] and increases in MMP9 correlate with ventricular enlargement and dysfunction after MI [114]. MMP9 promotes cardiac remodeling by ECM degradation and activation of a variety of chemokines and cytokines [115]. Based on ChIP-exo data for MEF2A in cardiomyocytes carried out in our lab [62], we identified common genes that are associated with MEF2A-binding events and those that are dysregulated at the mRNA level due to STAT3 depletion in the current study. Interestingly, this data comparison revealed that 10% of DEGs regulated by STAT3 are also bound by MEF2A. Of these genes, Tripartite motif-47 (TRIM47) is a well-characterized regulator of ischemia-reperfusion injury in cerebral tissue [116]. TRIM47 regulates apoptosis through activation of caspase-3 cleavage and pro-inflammatory factors through activation of nuclear factor-kappa B (NF- kB) signalling [116]. Another gene, encoding Glycoprotein Hormone Beta 5 (GBP5) is an inflammasome protein regulator that is implicated in caspase-1 dependent cell death and pyroptosis in diabetic cardiomyopathy [117]. Tescalcin (TESC) is a Ca^2+^-binding protein that belongs to the EF-hand superfamily and is expressed abundantly in the heart of adult mice. Tesc shares sequence and functional similarity with calcineurin-B homology protein (CHP). Based on that similarity, it is possible that TESC can inhibit the phosphatase activity of calcineurin-A, a well-established regulator of cardiomyocyte transcriptional regulation [118]. Interferon regulatory factor-2 (IRF-2) is a member of the interferon [119] regulatory factor family. IRF-2 plays a role in viral infection through the IFNs signalling regulation [120]. Zinc finger NFX-1 containing protein 1 (ZNFX1) belongs to a helicase superfamily 1 (SF1) which acts as a dsRNA sensor that restricts the replication of RNA virus and is a regulator of viral-induced immunity [121]. In Addition, human myocarditis is associated with an increase in the expression of another gene in this list, colony-stimulating factor1 (CSF-1). CSF-1 is responsible for the differentiation and maturation of monocytes that are involved in the inflammatory response-which induces cardiac damage during viral and autoimmune myocarditis [122].

Based on the integration of multiomic data we have carried out, one prevalent theme that emerges is the association of MEF2 and STAT3 with genes dynamically regulating the inflammatory response and cell survival. These observations suggest a complex interplay in transcriptional regulatory networks regulated by MEF2A and STAT3 that provide hierarchical control over these cellular processes. This idea explains why depletion of either of these molecules in cardiomyocytes leads to profound alterations in cell viability previously reported [48, 61]. In summary, we have carried out an affinity-based quantitative proteomics approach to characterize MEF2A interactome components in cardiomyocytes. This approach has identified a number of MEF2A interacting partners that will inform future studies concerning the hierarchical regulation of cardiomyocyte gene regulation. In particular, we have documented an interaction between MEF2A and STAT3 that may balance the regulation of critical gene networks associated with the inflammatory response, cardiomyocyte cell survival and cardiac hypertrophy.

## Materials and methods

### Primary cardiomyocyte preparation

Hearts were isolated from 1–3 days old Sprague Dawley rat. A two-step enzyme digestion is employed for the isolation using the Neonatal Cardiomyocyte Isolation System (Worthington Biochemical Corp). The whole hearts were isolated from sacrificed pups and digested with trypsin and collagenase. The isolated cells were re-suspended in DMEM/F12 (Gibco) containing 1% Penicillin/Streptomycin, 50 mg/L gentamycin sulphate), and 10% fetal bovine serum (FBS). Cells were pre-plated for 2 h at 37 °C with 5% CO_2_ to remove non-cardiomyocyte cell contamination. Cardiomyocytes were counted using a Hemocytometer, then seeded on gelatin-coated plates to recover overnight in F12/DMEM growth medium. The following day, the media was removed and replaced with fresh, serum-free media before subsequent experimentation. Animal studies were carried out in accordance with the Canadian Council of Animal Care (CCAC) mandate and guidelines.

### Plasmids and oligonucleotides

The expression plasmid for full-length pMT2 MEF2A, 4xMEF2-Luc and α-MHC-Luc reporter gene constructs have been described by us previously [41, 45, 123]. pCMV5-Flag-HDAC3 was a gift from Qingbo Xu and Lingfang Zeng (Addgene plasmid #63676). A luciferase reporter plasmid driven by 996 bp of the human MMP-9 promoter was a gift from Etty Benveniste & Douglas Boyd (Addgene plasmid # 53434). 2xSTAT-Luc was constructed by insertion of 2xGAS; 5′-TCGAGTCTTCCGGGAAATGCAGATCTTTCCGGGAAATGCAGAT (Sigma Genosys) into XhoI/Bg1II sites in front of a c-fos minimal promoter in the pGL3-basic luciferase reporter vector (Promega; USA). In order to construct the constitutively active version of STAT3-ß, we first constructed the expression plasmid for STAT3-ß by insertion of PCR amplified nucleotide of STAT3 ORF with Fwd-primer (5′-TAGAATTCATGGCTCAGTGGAACCAG) and Rev-primer (5′-ATCTCGAGTTATTTCCAAACTGCATCA), into EcorRI/XhoI site of pcDNA 3. Then, STAT3-ß, CA form was generated by site-directed mutagenesis of A662C and N664C using Fwd-primer (5′-GCTATAAGATCATGGATTGTACCTGCATCCTGGTGTCTCC) and Rev-primer (5′-GGAGACACCAGGATGCAGGTACAATCCATGATCTTATAGC). 3xFlag-MEF2A construct was cloned using Gateway technology (life Technology) into pDEST 5′ Triple Flag pCDNA5 FRT TO vector carrying an N-terminal 3xFlag tag and ccdb sequence between attR1 and attR2. PCR product for full-length MEF2A was generated with primers containing attB sites. The PCR reaction was cleaned using Qiagen PCR purification kit (Life Technologies– Cat #: 28104) and cloned into pDONR223 vector using the BP clones kit (Life Technology– Cat #: 11789). The entry clone was recombined into the pDEST 5′ Triple Flag pCDNA5 FRT TO destination vector using the LR clonase kit (Life Technologies– Cat#: 12538). The resulting vector was verified by DNA sequencing.

### Antibodies and reagents

Rabbit MEF2A polyclonal antibody were produced in house with the assistance of the Animal Care Facility at York University (Toronto, ON, Canada). The following antibodies were purchased from Santa Cruz: α-β actin (sc-47778), and α-RBPMS (sc-293285). The following antibodies were obtained from Cell Signaling: α-STAT3 (9139), P-STAT3 Tyr705 (9131 S), Goat α-mouse IgG-HRP (1706516) and goat α-rabbit IgG-HRP (1706515) were from Bio-Rad Laboratories. The following antibodies were purchased from Millipore sigma: α-Flag (F2555), normal rabbit IgG(2729 S), TRITC and FITC-conjugated α-mouse and α-rabbit secondary antibodies. Mouse MMP9 antibody (ab283575) was purchased from Abcam. STAT3 inhibitor XIII (C188-9 (573128)), HDAC3 inhibitor (RGFP966), Isoproterenol hydrochloride (1351005), and (R)-(-)-phenylephrine hydrochloride (P6126) were purchased from Sigma.

### Cardiomyocyte transfection

Primary cardiomyocytes (PCMs) were transfected with the 3xFlag-MEF2A construct using Lipofectamine2000 (Invitrogen). According to the manufacturer’s instructions, Lipofectamine2000 and 3xFlag-MEF2A plasmid were diluted separately into Opti-MEM medium, then mixed and incubated for 15 min at room temperature. The DNA/Lipofectamine mixture was added to the cells and incubated at 37 °C overnight. The media was then replaced and the cells were harvested 48 h later for analysis. For the siRNA experiment, PCMs were transiently transfected with siRNA (Sigma-Aldrich) using Lipofectamine RNAiMAX (Invitrogen) following the manufacturer’s instructions. Lipofectamine RNAiMAX reagent and siRNA (200 nM) were diluted into Opti-MEM medium in separate tubes, then mixed and incubated for 15 min at room temperature. The mixture was added to the PCMs and incubated at 37 °C overnight followed by replacement with fresh media. The cells were harvested 48 h later. The purchased siRNAs used in the study were siSTAT3#1 (SASI_Mm01_00041178), siSTAT3#2 (SASI_Mm01_00041179), siMEF2A#1 (SASI_Mm01_00120787), siMEF2A#2 (SASI_Mm01_00120788), and scrambled control (SIC001).

### Pharmacological treatment of primary cardiomyocyte cultures

To study the effect of HDAC3 on STAT3, the PCMs were treated with a HDAC3 inhibitor (RGFP966) (10 μM) for 24 h, DMSO was added to the media as the corresponding control. The PCM’s were treated with a p38 MAPK inhibitor (SB203580; 10 μM) or its inactive analogue (SB202474; 10 μM) for 24 h in serum-free media. To inhibit the STAT3 Y705 phosphorylation, a STAT3 inhibitor XIII, C188-9 (10 μM) was added to the media for 1 h before harvesting the cells. The control cells were treated with the solvent (DMSO). To induce hypertrophy in PCMs, Isoproterenol hydrochloride (Sigma Aldrich, 10 μM) and phenylephrine (Sigma‐Aldrich, 200 μM) were added to the media and PCMs were cultured for 24 h and 48 h before harvesting, respectively.

### Protein extraction and Western blot

Cells were harvested using NP-40 lysis buffer containing 0.5% (V/V) NP-40, 50 mM Tris-HCl [pH 8], 150 mM NaCl, 10 mM sodium pyrophosphate, 2 mM EDTA [pH 8], 0.1 M NaF, protease inhibitor cocktail (Sigma Aldrich), 1 mM phenyl methyl sulfonyl fluoride (Sigma Aldrich), and 1 mM sodium orthovanadate (Bioshop, Burlington, ON, Canada). Extracted proteins were denatured in SDS loading buffer at 100 °C for 5 min, electrophoretically run in 8 or 10% SDS-polyacrylamide gels, transferred into Immobilon-FL polyvinylidene difluoride membrane (Millipore). Non-specific binding was blocked with 5% skim milk in Tris-buffered saline (TBS)-T (10 mM Tris-HCl, pH 8.0, 150 mM NaCl, 0.1% Tween 20) for 1 h prior to antibody incubation. Membranes were incubated overnight with primary antibodies at 4 °C in blocking buffer. Primary antibodies included MEF2A (1:500), STAT3 (1:1000), Flag (1:1000) and actin (1:2000). The blots were then incubated with the appropriate horseradish peroxidase-conjugated secondary antibodies as follows: anti-rabbit (1:2000) and anti-mouse (1:2000) for 2 h at room temperature. Protein was detected by Enhanced Chemiluminescence (ECL) western blotting substrate (Pierce, ThermoFisher). Full and uncropped western blots are presented in supplemental file.

### Immunoprecipitation

Non-transfected and 3xFlag-MEF2A transfected PCMs were harvested and proteins were extracted as previously mentioned. Immunoprecipitation (IP) was performed using anti-Flag M2 magnetic beads (MilliporeSigma) and ImmunoCruz Optima kit (Santa Cruz Biotechnology) according to the manufacturer’s instructions. Eluted proteins were analyzed by Western blotting as described previously.

### Sample preparation for mass spectrometry

Six immunoprecipitates were processed (3 from Flag-MEF2A expressing and 3 from control non-transfected PCMs) using anti-Flag magnetic beads. Immunoaffinity-captured Flag-tagged fusion proteins, along with the subset of endogenous proteins bound to them, were eluted by rapid pH drop achieved by the addition of 200 μL of 0.2% TFA in 20% acetonitrile (pH 1.9) for each sample (100 μL 2X, each time for 10 min). Each eluate fraction gave rise to material for for mass spectrometric analyses (180 μL) and western blotting (20 μL). The mass spectrometry samples, containing eluates of purified Flag-MEF2A and its associated factors, were then trypsin-digested and modified by isobaric labelling (iTRAQ, Sciex), followed by LC-MS/MS for peptide identification and quantification (Fig. 1B) as described previously [124].

### iTRAQ quantitative mass spectrometry

The iTRAQ-modified tryptic digests were mixed, then desalted on C18 microcolumns (Agilent, Santa Clara, USA, product number A57003MB) and reversed phase fractionated (Thermo, product number 84868) in 0.1% triethylamine, applying step elutions in the presence of 12.5, 17.5, 22.5 and 50% acetonitrile. Subsequently, fractions were dried in a centrifugal concentrator, then resuspended in aqueous 0.1% formic acid (v/v) and injected for online nano-LC separation on a 25 cm long, 75 micrometer wide C18 column packed with a stationary phase composed of 2 micrometer beads featuring pores of 100 Angstrom (Thermo, product number 164941, Waltham, USA). Chromatographic separation over an acetonitrile gradient was delivered via Easy nLC-1000 (Thermo) that was online coupled by electrospray ionization to an Orbitrap Fusion ETD mass spectrometer (Thermo). The latter operated on a continuous 3-second cycle encompassing a precursor ion orbitrap scan, followed by MS2 and synchronous precursor selected MS3 scans of the 10 most abundant precursor ions on the linear ion trap. Dynamic precursor ion exclusion operated over a mass range of 20 ppm and was maintained over a 100-minute acquisition time window to limited redundancy in MS2-MS3.

Peptide sequence and abundance information was extracted from raw LC-MS data by Proteome Discoverer software (Version 1.4, Thermo) housing Sequest HT, with a fixed false discovery rate of 5%. The UniProt rat proteome was used as a sequence reference. The Reporter Ions Quantifier was operated at a m/z tolerance of 20 ppm.

All identified proteins were quantified in each sample. Because the iTRAQ quantitation produces relative quantitations, protein levels are presented as median abundance ratios (control-over-control and MEF2A-over-control). One control sample “Control-3” was used as a common denominator and the other five samples (MEF2A 1, MEF2A 2, MEF2A 3, Control-1, Control-2), were all compared relative to “control 3”. Abundance ratios are are colour-coded to reflect the magnitude of the change (red for less than 1 and blue for greater than 1). Based on a qualitative assessment of the dataset, we established criteria that a MEF2A interactor should be expected to have MEF2A/Control 3 ratios of at least 2 for all three MEF2A replicates and Control/Control-3” ratios of 2 or less for both control/control ratios. The 56 most co-enriched proteins are shown in Table 1 supplementary document 1.

### Immunofluorescence

PCMs were seeded onto glass-bottom dishes (MatTek Corp). The cells were transfected with expression vectors for Flag-MEF2A and Myc-STAT3b. Before imaging the cells, the media was replaced for DMEM/F12 (Gibco). Hoechst 33342 (Sigma-Aldrich) was added to 2.5 μM into the media to stain nuclei. After 30 min, the stained cells were visualized using a Carl Zeiss Spinning disc system (Zeiss Observer Z1 with Yokogawa CSU-X1 and Axio- Cam MRm camera) in the environment chamber (37 °C, 5% CO_2_). The raw images were processed using ZEN software (Carl Zeiss) to obtain pseudo-colored micrographs.

### Reporter gene assays

PCMs were washed three times with 1xPBS and then harvested by Luciferase lysis buffer containing 20 mM Tris pH 7.4 and 0.1% Triton X-100. The enzymatic activity in the lysates was measured using a Lumat LB (Berthold) luminometer after addition of Firefly Luciferase assay substrate (E1501, Promega) or Renilla Luciferase assay substrate (E2820, Promega). Firefly Luciferase values were normalized to the corresponding Renilla luciferase activity to normalize for transfection efficiency and the values were expressed as fold activation over corresponding control conditions.

### Sample preparation for RNA sequencing

First, total RNA was isolated from the PCMs using Qiagen RNeasy Plus Mini Kit (cat. nos. 74134). The concentration and quality of isolated RNA was assessed using a NanoDrop spectrophotometer. ThermoFisher Low Input RiboMinus Eukaryote Kit (cat. No. A15027) was used to remove rRNA from the extracted RNA. Illumina NGS library was prepared with the Nextera XT DNA library preparation kit (Illumina). Paired reads were generated using Illumina NextSeq 500 (151 bp read length).

### Differential expression analysis of RNA transcripts in primary rat cardiomyocytes

Raw counts were analyzed with the non-alignment (kmer-based) computational software kallisto [125]. The main output of this tool are the estimated read counts and TPM (transcripts per million) values for 31715 transcripts isoforms (with Ensemble rat rn6 isoform IDs of the type ENSRNOT00000047550.4). The differential expression analysis was conducted edgeR [126] with the TPM values [127].

### Bioinformatics analysis

To further interrogate the MEF2A interactome, bioinformatics tools were employed to retrieve information on protein annotation, protein-protein networks, and potential pathways. A Gene Ontology (GO) analysis of ‘biological process’, ‘cellular component’, and ‘molecular function’ was performed using DAVID software (http://www.david.niaid.nih.gov) to indicate enrichment GO functions. The String database (https://string-db.org) and Cytoscape software (https://cytoscape.org) were used to visualize protein-protein interaction networks. Protein networks were further interrogated using Ingenuity Pathway Analysis (IPA, http://www.ingenuity.com/) to identify interaction networks and predominant canonical pathways associated with the MEF2A interactome and theRNAseq and ChIPseq datasets. The scores associated with each form of analysis were calculated using a logarithm of the p-value (Fisher’s exact test), indicating the chance that the proteins found in a given network would be false.

### Statistical analysis

Data are reported as means ± SEM. All data were verified in three or four independent biological replicate experiments using different batches of cardiomyocyte isolates. Independent two-sample *t*-tests of all quantitative data were conducted, whereas a one-way analysis of variance followed by a Tukey HSD post hoc test or Dunnett’s multiple comparisons test (as indicated in figure legends) were performed on experiments with greater than two experimental conditions. *P*-values are indicated with respect to controls where appropriate and *P* < 0.05 was considered statistically significant.

## Supplementary information

Supplemental Document 1

Supplemental Document 2

Supplemental Document 3

SUPPLEMENTAL MATERIAL

Western Blots

Checklist

## Data Availability

The materials and reagents described herein are freely available to the research community to use for non-commercial purpose. RNA-seq data for STAT3 depletion described herein are deposited in NCBI SRA with Bioproject accession PRJNA930698. The mass spectrometry proteomics data (MEF2A interactome) have been deposited to the ProteomeXchange Consortium via the PRIDE partner repository with the dataset identifier PXD038574.
