# Supplementary material for: The MEF2A transcription factor interactome in cardiomyocytes
Source: Cell Death Dis. 2023 Apr 5;14(4):240. doi: 10.1038/s41419-023-05665-8 (PMC10076289; doi:10.1038/s41419-023-05665-8)
Supplement: Supplementary file 4 — SUPPLEMENTAL MATERIAL [file 41419_2023_5665_MOESM4_ESM.pdf]

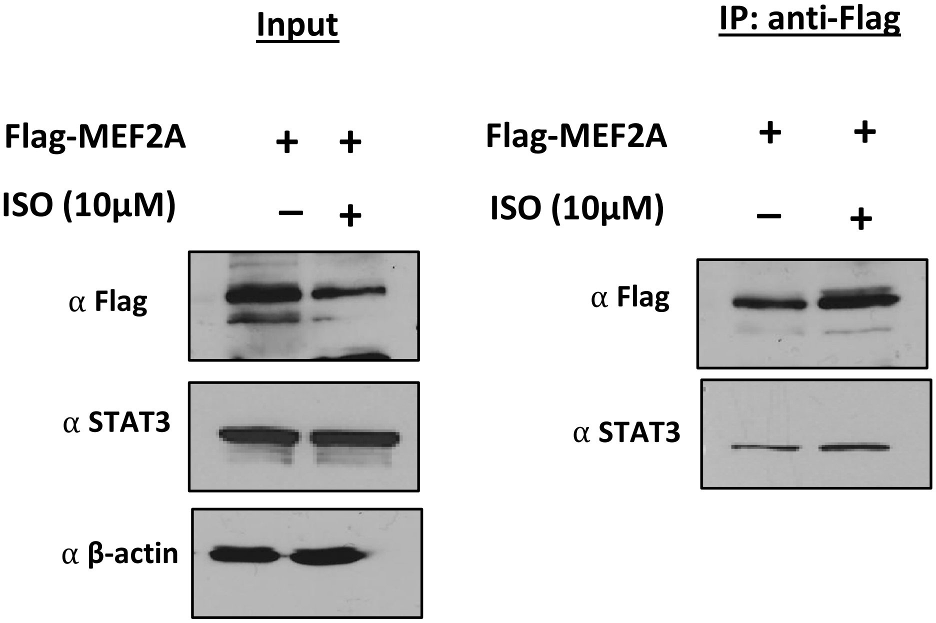

Figure S1. PCMs were transfected by Flag-MEF2A construct for 48h. PCMs then treated by ISO (10μM) and and vehicle (H2O) for 24 h. Lysates were assessed for overexpression by western blot. Flag-MEF2A lysates were used for IP using a-Flag magnetic beads and the eluates were blotted with Flag and STAT3 antibodies. Number of biological replicates for western blot and IP are three.

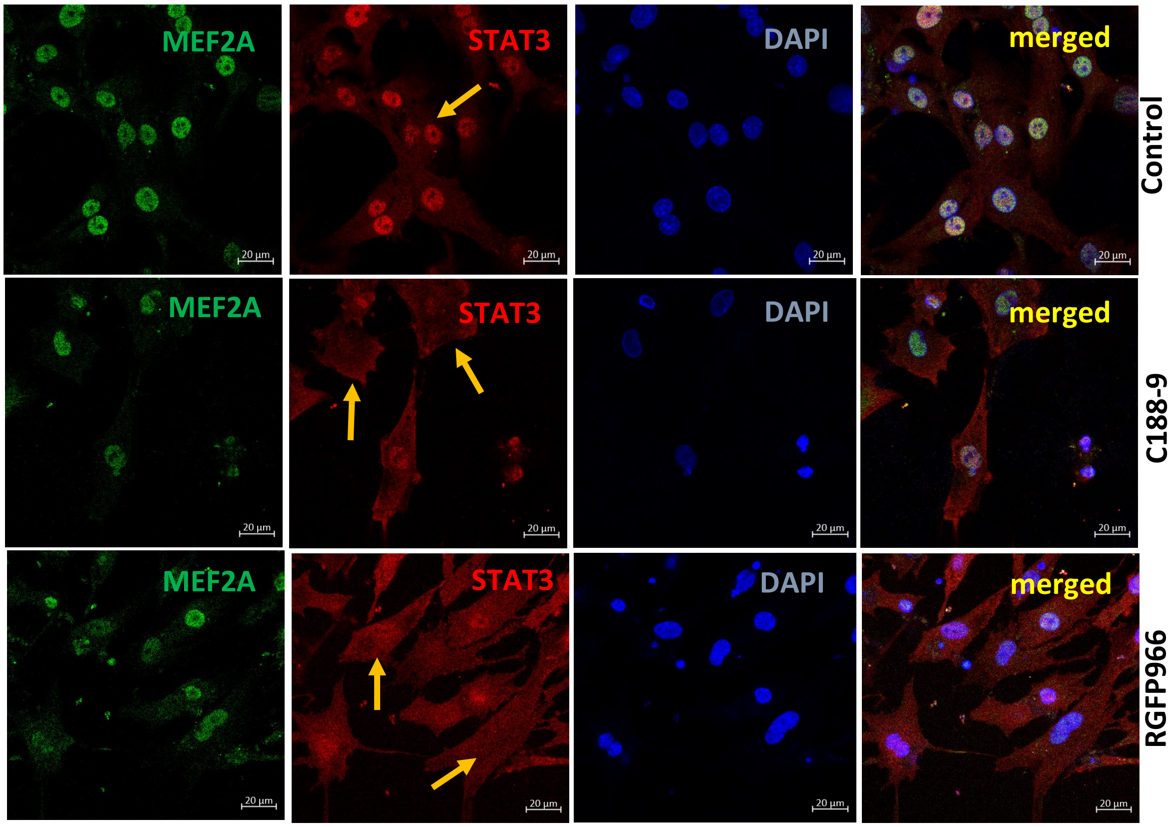

Figure S2. Confocal immunofluorescence analysis of endogenous MEF2A and STAT3 shows a nuclear localization upon STAT3 inhibitor XIII, C188-9 (10µM) for 1 h and HDAC3 inhibitor (RGFP966) (10µM) for 24 h PCMs. PCMs were fixed and stained for MEF2A in green and counter stained for STAT3 in red, DAPI in blue. Slides were analyzed by confocal microscopy in separate green, red, and blue channels and in a merged image. The scale bar is 20µm.

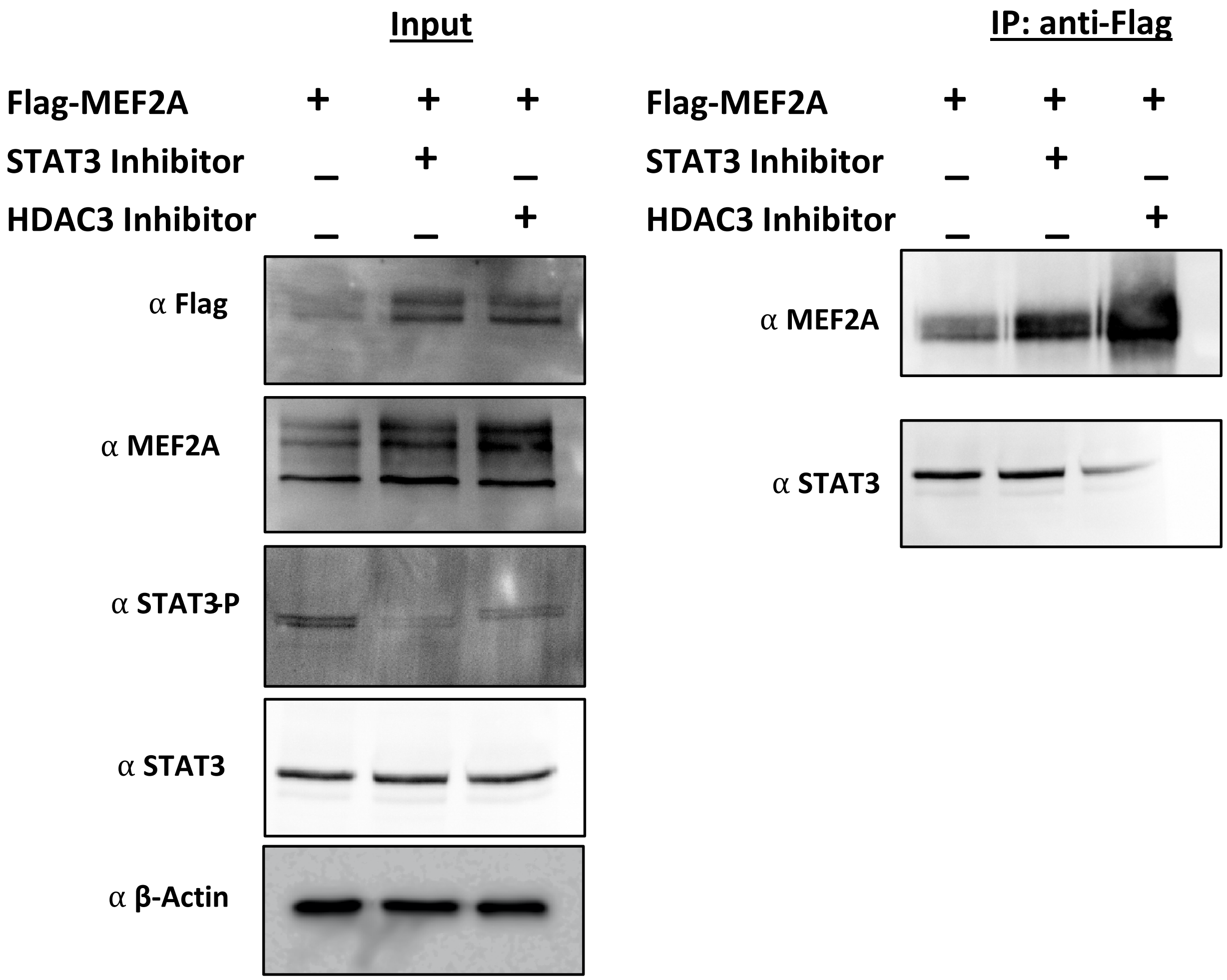

Figure S3. PCMs were transfected by Flag-MEF2A construct for 48h. PCMs then treated by HDAC3 inhibitor (RGFP966) (10µM) for 24 h and STAT3 inhibitor XIII, C188-9 (10µM) for 1 h. Lysates were assessed for overexpression and protein levels by western blot. Flag-MEF2A lysates were used for IP using a-Flag magnetic beads and the eluates were blotted with MEF2A and STAT3 antibodies. Number of biological replicates for western blot and IP are three.
