# Supplementary material for: The MEF2A transcription factor interactome in cardiomyocytes
Source: Cell Death Dis. 2023 Apr 5;14(4):240. doi: 10.1038/s41419-023-05665-8 (PMC10076289; doi:10.1038/s41419-023-05665-8)

Fig:1C Uncropped

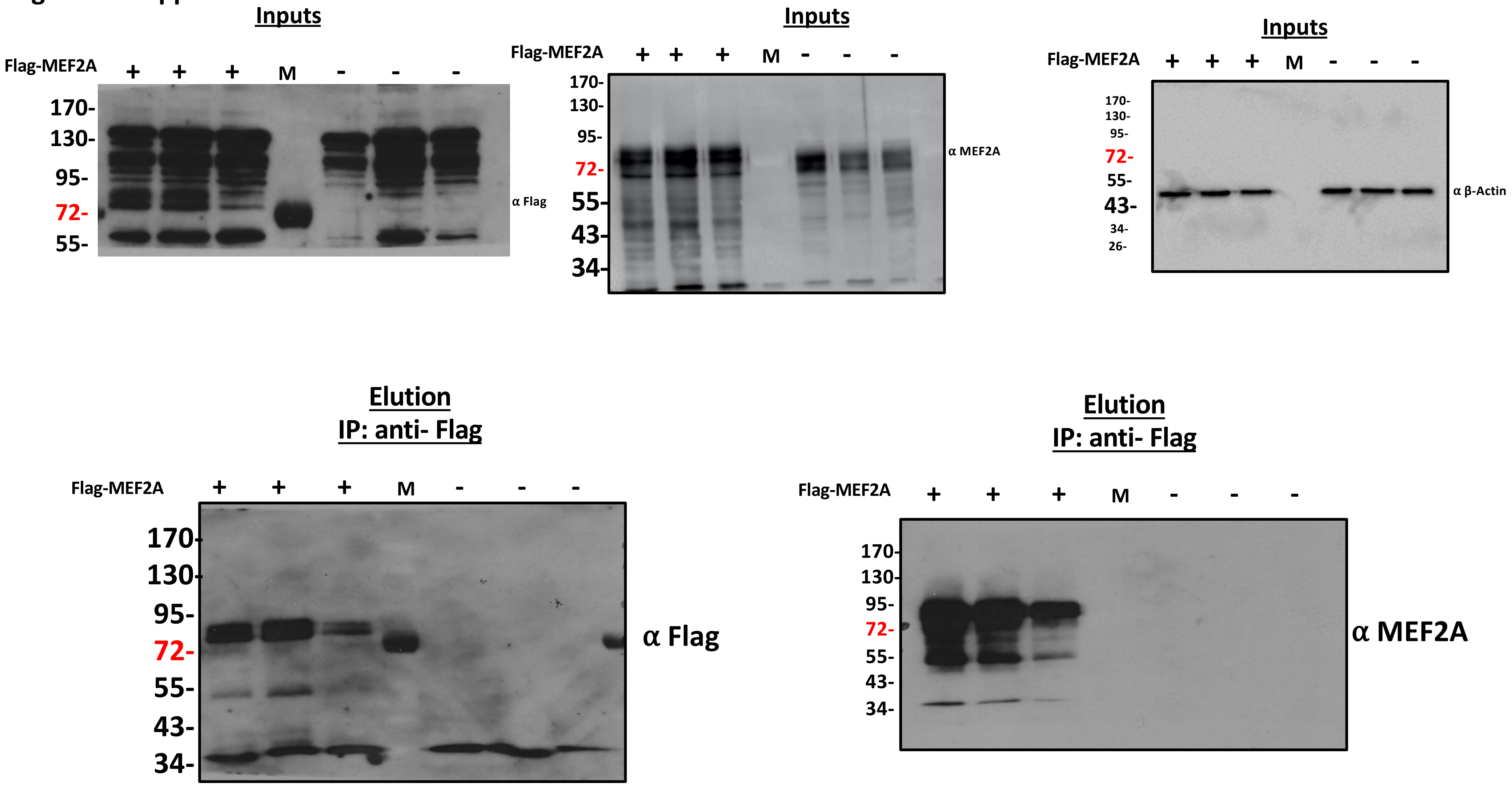

Fig:3A Uncropped

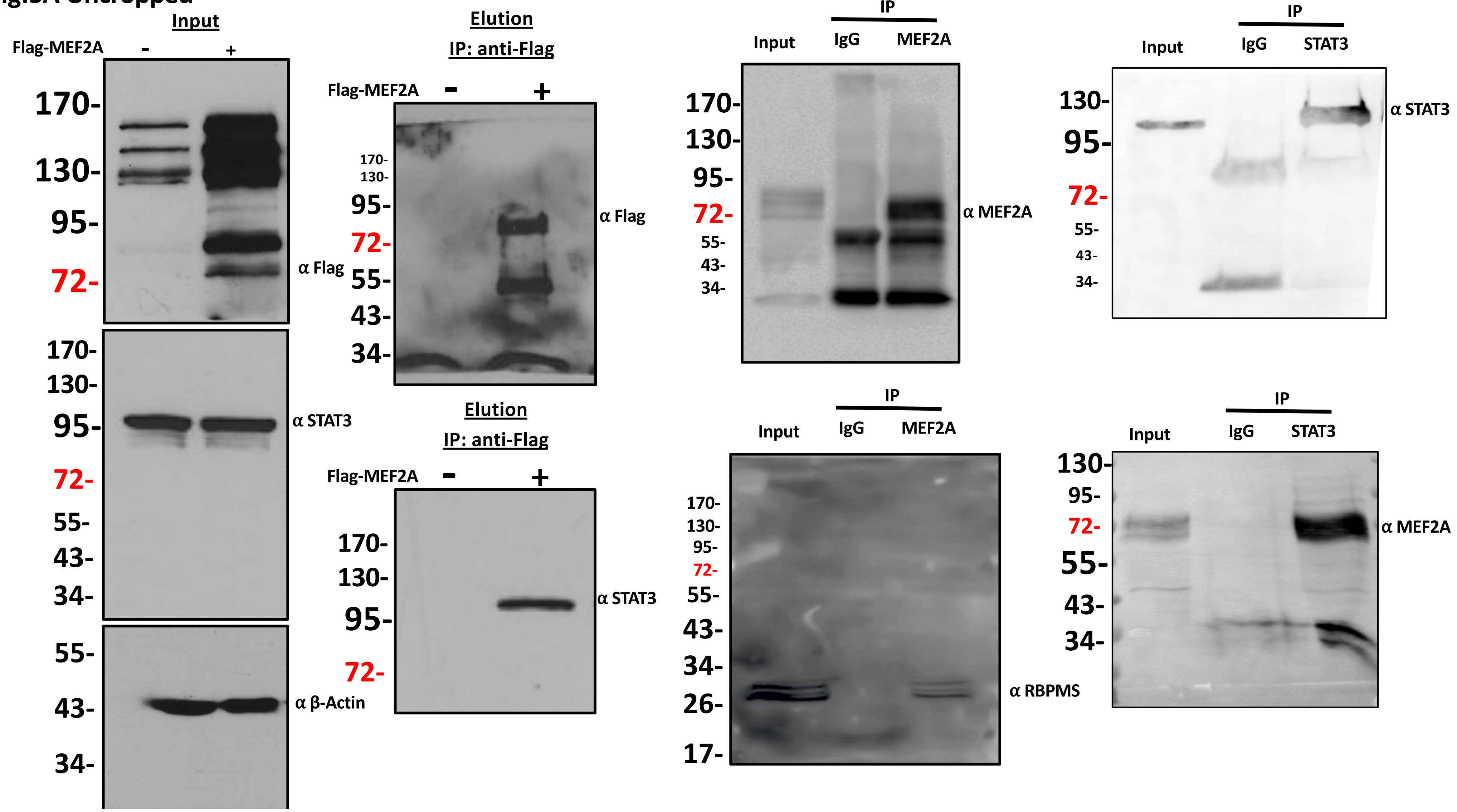

Fig:3D Uncropped

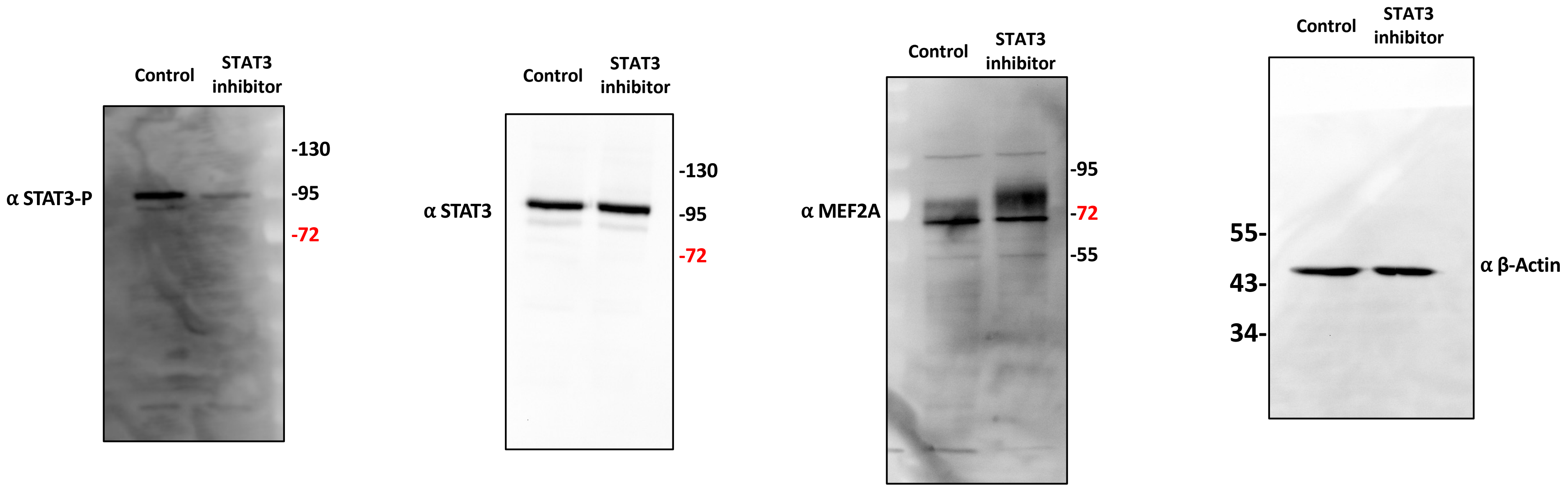

Fig:4A Uncropped

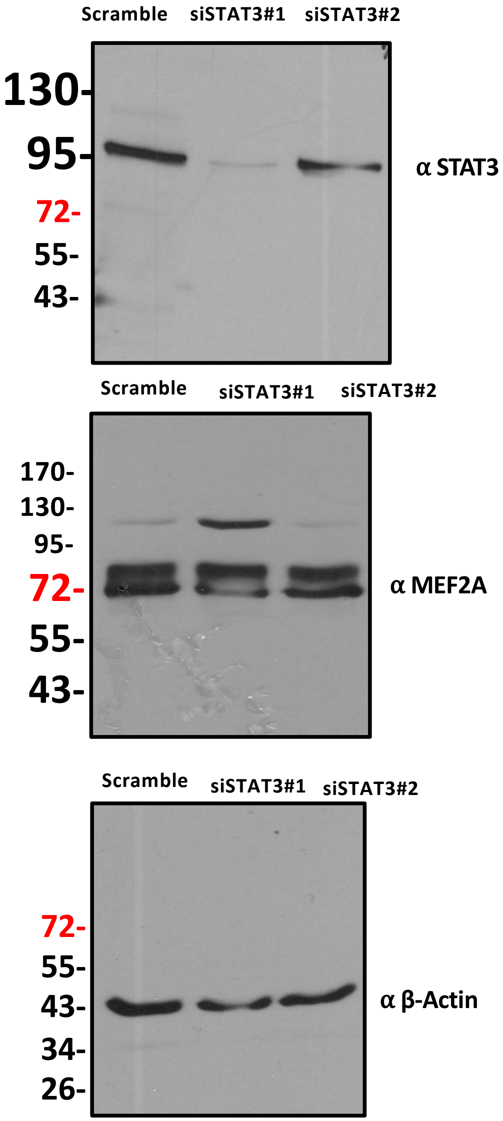

Fig:4B Uncropped

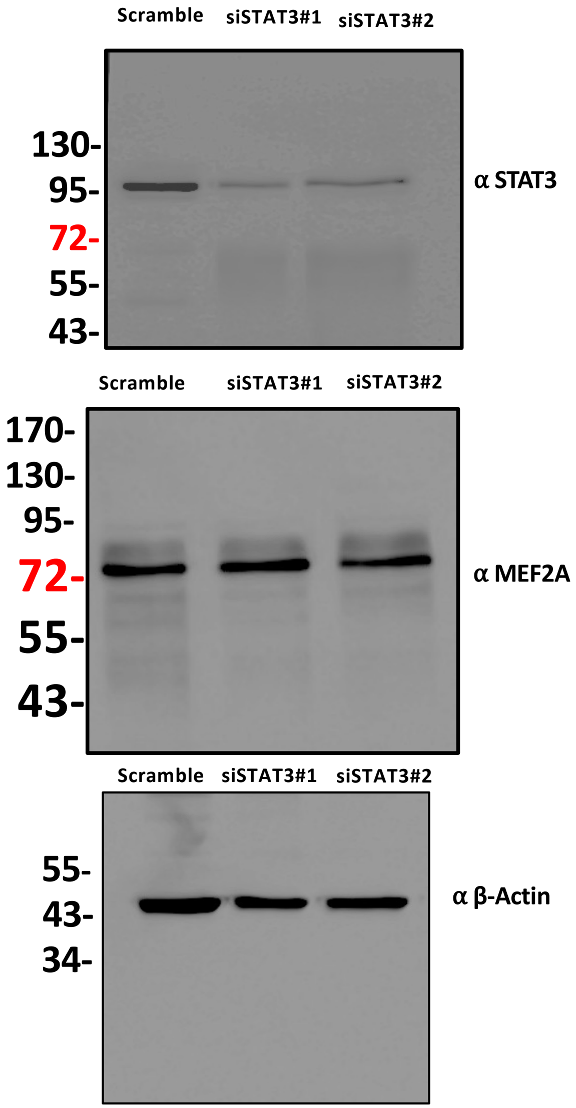

Fig:4C Uncropped

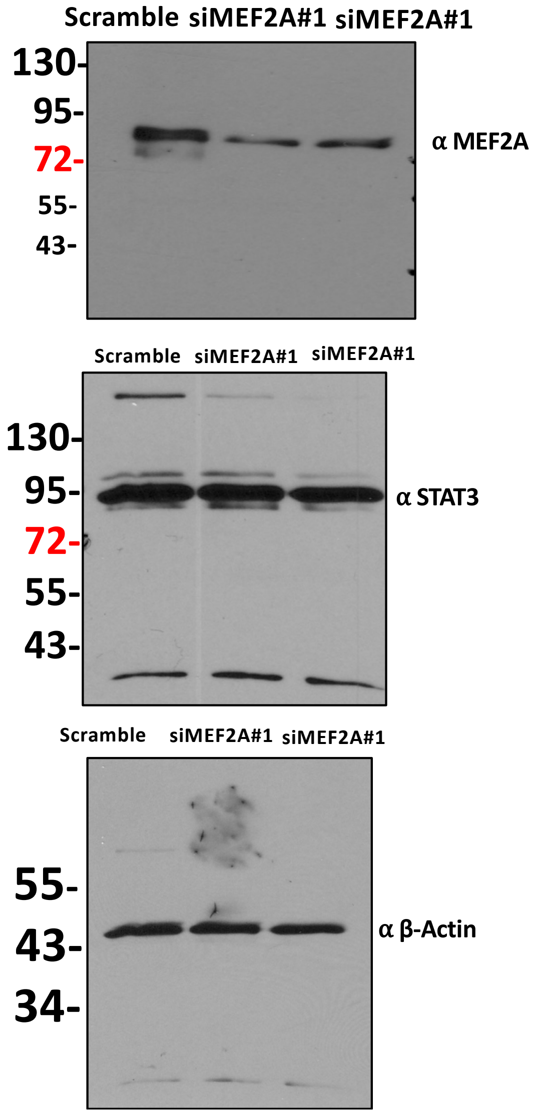

Fig:4D Uncropped

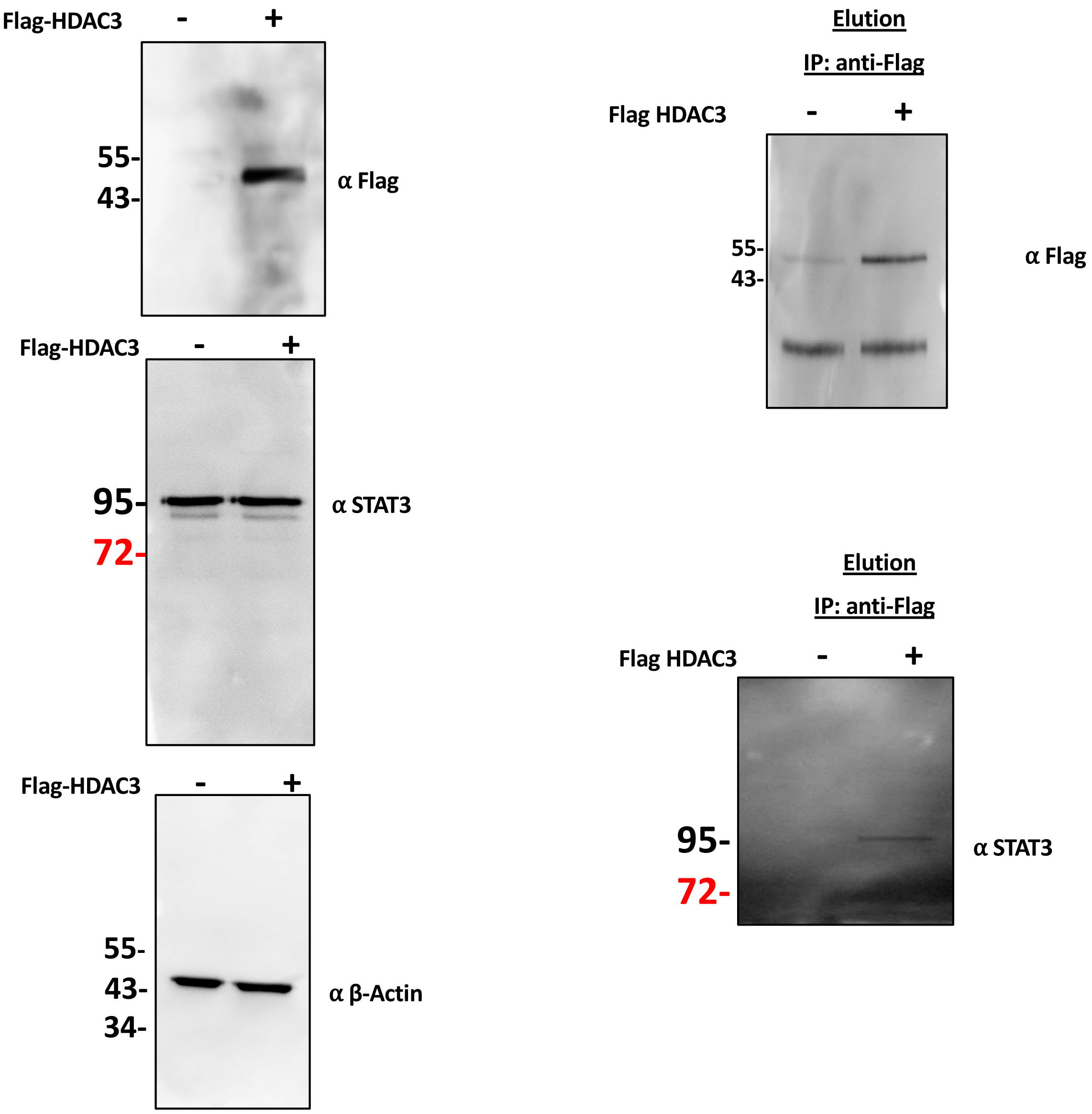

Fig:5B Uncropped

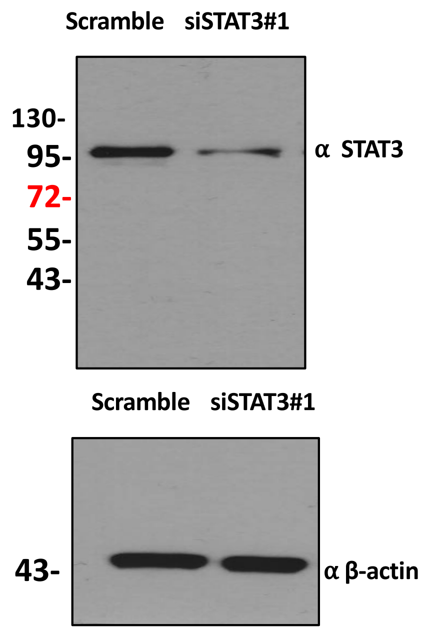

Fig:7A Uncropped

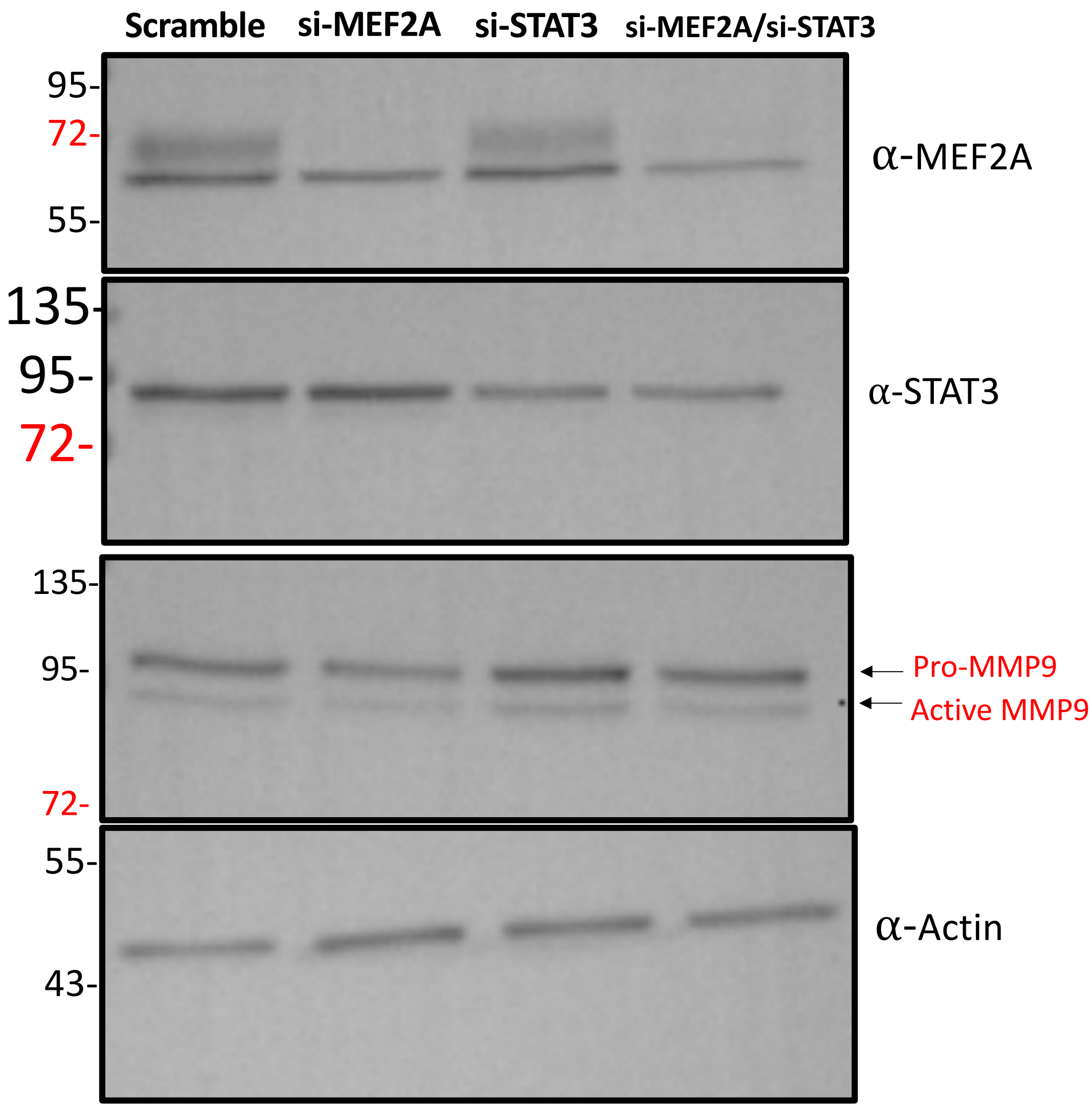

Fig:7B Uncropped

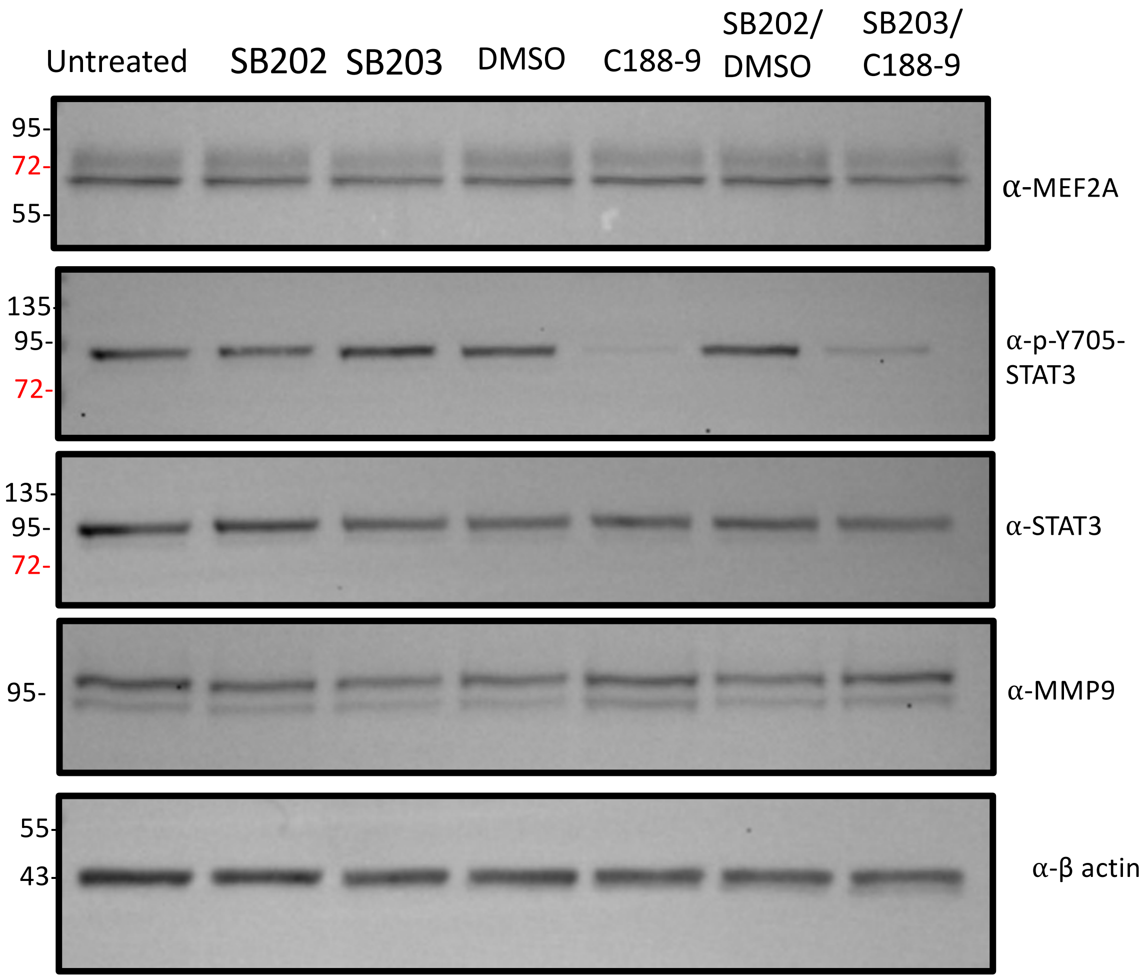

Fig:S1 Uncropped

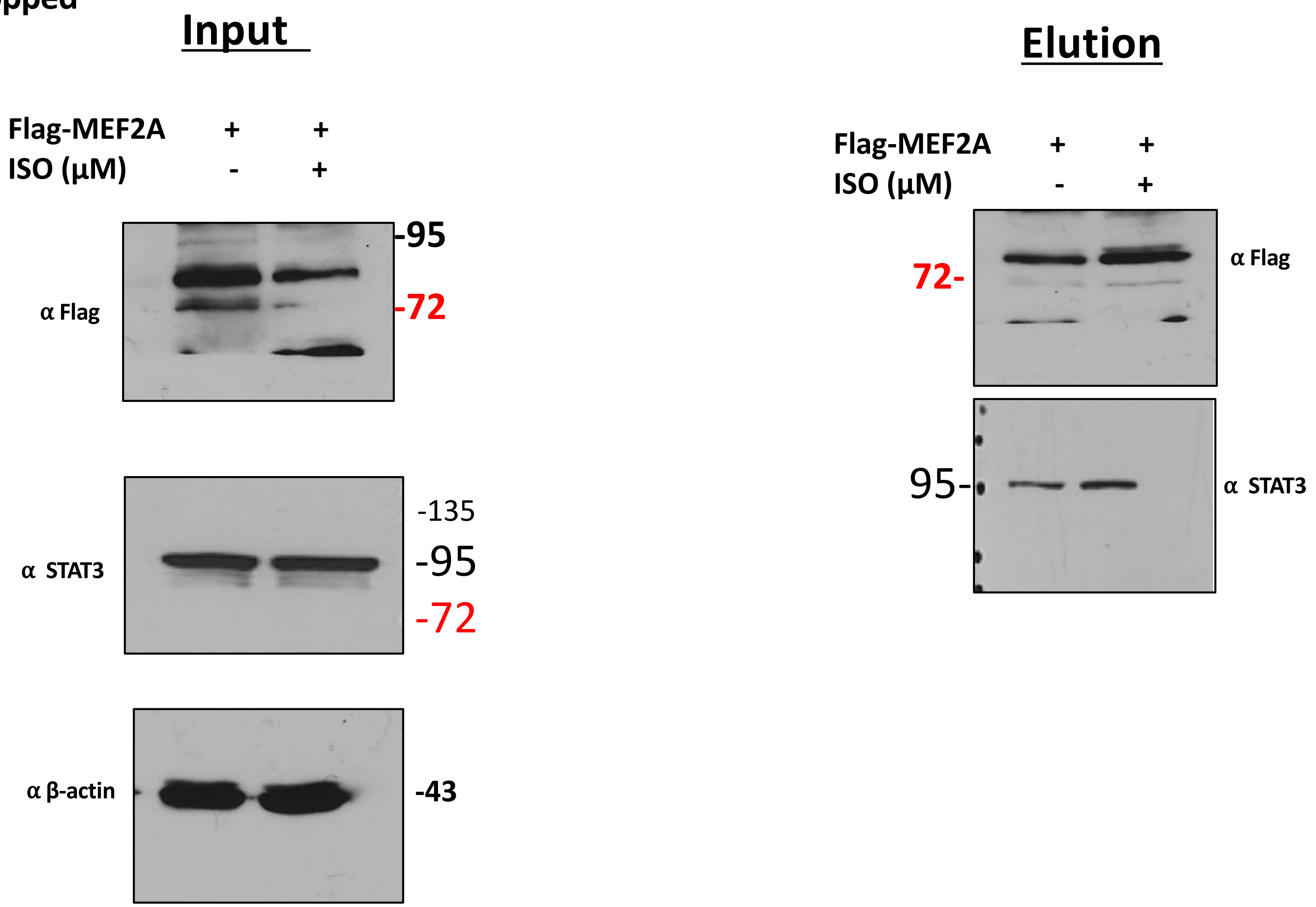

Fig:S3 Uncropped

Inputs

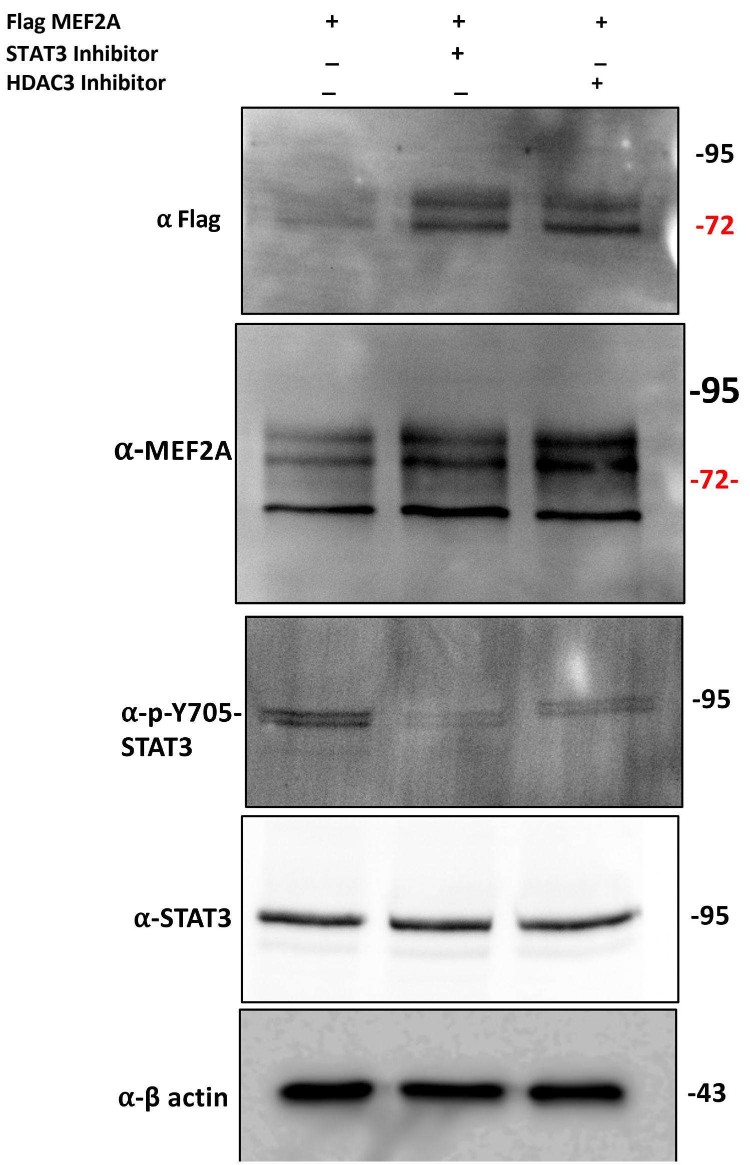

Elution

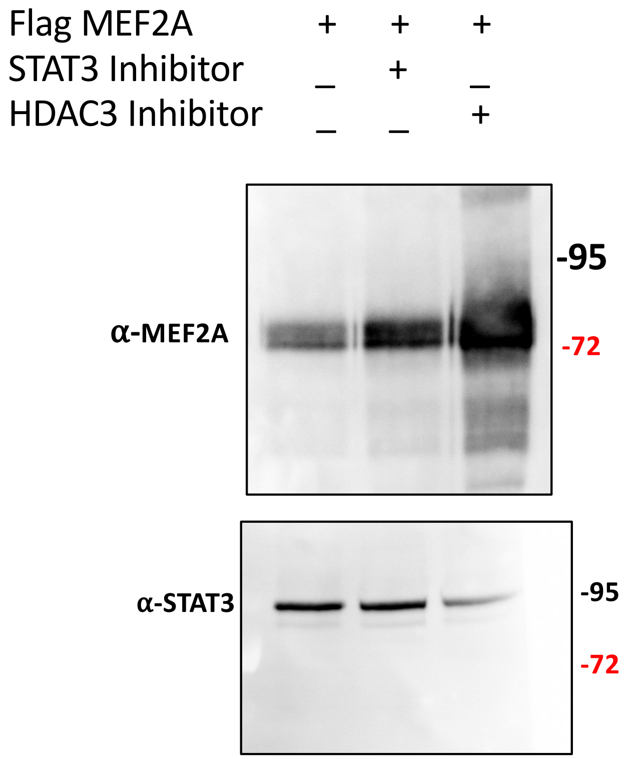

Supplement: Supplementary file 5 — Western Blots [file 41419_2023_5665_MOESM5_ESM.pdf]
